# Application of machine learning in predicting non-alcoholic fatty liver disease using anthropometric and body composition indices

**DOI:** 10.1038/s41598-023-32129-y

**Published:** 2023-03-27

**Authors:** Farkhondeh Razmpour, Reza Daryabeygi-Khotbehsara, Davood Soleimani, Hamzeh Asgharnezhad, Afshar Shamsi, Ghasem Sadeghi Bajestani, Mohsen Nematy, Mahdiyeh Razm Pour, Ralph Maddison, Sheikh Mohammed Shariful Islam

**Affiliations:** 1 Department of Nutrition, Faculty of Medicine, Hormozgan University of Medical Sciences, Shahid Chamran Boulevard, Bandar Abbas, Iran; 2 Institute for Physical Activity and Nutrition (IPAN), Deakin University, Geelong Victoria, Australia; 3 Department of Nutrition, School of Nutrition Sciences and Food Technology, Kermanshah University of Medical Sciences, Kermanshah, Iran; 4 Institute for Intelligent Systems Research and Innovation (IISRI), Geelong Waurn Ponds Victoria, Australia; 5 Biomedical Machine Learning Lab, University of New South Whales, Sydney, Australia; 6 Concordia Institute for Information Systems Engineering, Concordia University, Montreal, Canada; 7 Department of Biomedical Engineering, Faculty of Engineering, Imam Reza International University, Mashhad, Iran; 8 Metabolic Syndrome Research Center, Faculty of Medicine, Mashhad University of Medical Sciences, Mashhad, Iran; 9 Department of Electronic Learning, Shiraz University, Shiraz, Iran

**Keywords:** Non-alcoholic fatty liver disease, Diagnostic markers

## Abstract

Non-alcoholic fatty liver disease (NAFLD) is the most common chronic liver disease, which can progress from simple steatosis to advanced cirrhosis and hepatocellular carcinoma. Clinical diagnosis of NAFLD is crucial in the early stages of the disease. The main aim of this study was to apply machine learning (ML) methods to identify significant classifiers of NAFLD using body composition and anthropometric variables. A cross-sectional study was carried out among 513 individuals aged 13 years old or above in Iran. Anthropometric and body composition measurements were performed manually using body composition analyzer InBody 270. Hepatic steatosis and fibrosis were determined using a Fibroscan. ML methods including k-Nearest Neighbor (kNN), Support Vector Machine (SVM), Radial Basis Function (RBF) SVM, Gaussian Process (GP), Random Forest (RF), Neural Network (NN), Adaboost and Naïve Bayes were examined for model performance and to identify anthropometric and body composition predictors of fatty liver disease. RF generated the most accurate model for fatty liver (presence of any stage), steatosis stages and fibrosis stages with 82%, 52% and 57% accuracy, respectively. Abdomen circumference, waist circumference, chest circumference, trunk fat and body mass index were among the most important variables contributing to fatty liver disease. ML-based prediction of NAFLD using anthropometric and body composition data can assist clinicians in decision making. ML-based systems provide opportunities for NAFLD screening and early diagnosis, especially in population-level and remote areas.

## Introduction

Non-alcoholic fatty liver disease (NAFLD)‒ the hepatic manifestation of metabolic syndrome‒ is the most common chronic liver disease^1,2^. Worldwide prevalence of metabolic syndrome and NAFLD has increased in parallel with increased obesity prevalence^3–5^, which is about 20–30% in developed countries and one-third among American adults^6–8^.

Obesity is a common metabolic risk factor associated with NAFLD^9–11^. The prevalence of NAFLD is directly related to increased body mass index (BMI) and central obesity^12–14^. Most studies have shown that visceral fat is an independent factor in generating hepatic steatosis, independent of BMI^15,16^. The amount of adipose tissue and its distribution differs between men and women^17^. Women have higher overall fat tissue with relatively more subcutaneous adipose tissue in the hips and thighs. At the same time, men accumulate visceral and subcutaneous fat mainly in the trunk and abdomen with continuous changes before and after puberty^17–19^. The increased fat distribution around the waist (i.e. apple-shaped body) is linked to NAFLD in both genders^20^. In a pear-shaped body, the subcutaneous fat accumulates mainly in the thighs and buttocks^21,22^, which is typical among females but can increase metabolic syndrome in males, which is a risk factor for NAFLD independent of central obesity^23^. In support of the role of fat distribution and anthropometric measures in NAFLD, studies have found several contributing factors, including abdomen circumferences, waist, neck and fat accrual in trunk and arms^24–29^.

Most people with NAFLD, including both children or adults, do not have differential symptoms at the early stages of the disease^30^. Notably, after the development of cirrhosis, different symptoms such as *caput medusa*, spider angioma, palmar erythema, ascites, and jaundice appear^31^. Therefore, early diagnosis is critical to prevent severe complications.

Ultrasonography and laboratory tests are typical diagnostic methods for detecting fatty liver disease. Ultrasound technique has relatively high accuracy in detecting the moderate-to-severe steatosis level and lower accuracy in earlier stages of NAFLD^32^. Notably, hepatic fibrosis cannot be diagnosed by ultrasonography^14,33^. Although typically used to detect fatty liver disease, laboratory tests are not useful for all ages and gender groups due to low accuracy^34,35^. Therefore, a precise, cost-effective, and non-invasive method to analyze symptoms of various stages of the fatty liver for NAFLD diagnosis is desirable. Such an approach is important to help with early diagnosis of NAFLD, which could help prevent hepatic steatosis progression to fibrosis, advanced cirrhosis, and hepatocellular carcinoma.

In recent years, machine learning (ML) models have been used as a novel approach in predicting NAFLD^36–39^. However, all of these studies have focused mainly on laboratory outcomes and have not considered body composition and anthropometric factors. Therefore, the primary aim of this study is to identify essential ML classifiers of NAFLDs using body composition and anthropometric indices. The secondary aim is to identify feature contributions to the prediction of NAFLDs.

## Materials and methods

### Study design and participants

This cross-sectional study was conducted to explore NAFLD phenotypes based on body composition and anthropometric indices. Participants were recruited from the eastern (Khorasan Razavi) and southern (Hormozgan) provinces of Iran, through advertisement on the notice boards of the university clinics, as well as via phone or email contact to potential participants. A total of 593 individuals aged above 13 years old were initially recruited. Eighty individuals were excluded from the study and 513 participants were remained. Exclusion criteria were as follows: the presence of underlying liver disease, taking medications (anti-hypertensive and anti-arrhythmic, anti-glycaemic, corticosteroids, nervous system agents, chemotherapy, Methotrexate and Tamoxifen), alcoholic patients with more than twice-a-week consumption, previous history of any type of cancer during the last year, history of surgery during the last 6 months, pregnant women. The study was conducted following the Declaration of Helsinki, and ethical approval was granted by Mashhad University of Medical Sciences following (Code: IR.MUMS.fm.REC.1395.64). All participants provided written informed consent. Also, written informed consent was obtained from guardians of participants aged under 18 years.

### Data collection

At each medical clinic, eligibility, demographics questionnaire, anthropometric, and body composition measurements were assessed by two trained nutritionists. Medical examination and disease diagnosis were performed by a general physician and an internal specialist, respectively. Demographic information, including sex, age, education, disease history and medications, were assessed by researcher using a questionnaire. Weight was measured using a digital weighing scale (Seca 704; Hamburg, Germany), height was measured using a wall height chart, and the body composition measures were assessed using InBody 270 (Inbody Co. Ltd, South Korea) body analyzer to measure per cent (%) body fat, total fat mass, muscle mass, as well as fat mass in the right/left leg, right/left arm and trunk with light clothing and without shoes. The circumferences of neck, chest, arm, wrist, waist, hips, abdomen, thighs, and length of ulna and leg were measured using a flexible tape measure with an accuracy of 0.1 cm. BMI was calculated by dividing weight (kilograms) by the height in meters squared^26^. Subcutaneous fat in the area below the scapula, arms biceps and triceps and the upper iliac crest was measured using a Saehan calliper (Saehan SH5020, Korea). Participants were also examined for acanthosis in the back of the neck and armpits and the presence of subcutaneous fat under the chin and at the back of the neck. A Fibroscan equipped with the M and XL probes (Echosens 504, Paris, France) was used to assess both controlled attenuation parameter (CAP) (dB/m) and liver stiffness measurement (LSM) (kPa) values simultaneously. A reliable LSM was defined as the median liver stiffness of the 10 measurements (a success rate of greater than 60%, and an IQR < 30% of the median LSM value)^40^. CAP values range from 100 to 400 dB/m and the following cut-off values were used for the diagnosis of steatosis stages: Stage 0, < 238 dB/m, Stage 1, ≥ 238 to 260 dB/m, Stage 2, ≥ 260 to 292 dB/m, and Stage 3, ≥ 292 dB/m^41^. LSM values range from 1.5 to 75 kPa, and the following cut-off values were used for the diagnosis of liver fibrosis stages: no significant fibrosis or F0 < 6.2 kPa, mild fibrosis or F1 ≥ 6.2 to 7.6 kPa, moderate fibrosis or F2 ≥ 7.6 to 8.8 kPa, severe fibrosis or F3 ≥ 8.8 to 11.8 kPa and cirrhosis or F4 ≥ 11.8 kPa^42^.

### Statistical analysis

Descriptive and non-predictive data analysis was performed using SPSS version 21 software (SPSS, Inc., Chicago, IL). Data were expressed as mean ± standard deviation or frequencies. Between-group comparisons were performed using an independent sample *t* test and analysis of variance (ANOVA), followed by Tukey’s post hoc test. A P-value of less than 0.05 was considered statistically significant.

### Machine learning models

Three label variables were considered: fatty liver (stage I, II and III vs. no steatosis), steatosis, and fibrosis stages. Eight ML techniques were applied to the dataset to identify the best modelling approach. To this end, k-Nearest Neighbor (kNN), Support Vector Machine (SVM), Radial Basis Function (RBF) SVM, Gaussian Process (GP), Random Forest (RF), Neural Network (NN), AdaBoost and Naïve Bayes were tested. An extant explanation of these classifiers can be found elsewhere^43^. Testing of these models was performed using the Scikit-learn library in Python programming language^44^.

To comprehensively compare different classifiers, we trained and evaluated dataset 50 times. This is because different classifiers sometimes predict slightly different outputs and initial points are different for a specific classifier in each run. Thus, a reliable output can be estimated by averaging each classifier several times. Model accuracy and area under the curve (AUC) are reported for each ML technique. Importance values are reported for individual feature variables.

Pre-processing involved data normalization and segmentation. The few missing values in the numerical results of the experiments were replaced using the Linear Interpolation method^45^. Principal component analysis (PCA) was used to extract the attribute of the data^46,47^. Data were divided into two parts, train and test. Processing involved feature selection and classification with the best feature. The model processing involved a variety of models. The model with the highest performance was selected.

### Patient consent

All patients provided written consent for participation in this study. For participants aged under 18 years, written informed consent was obtained from their guardians.

### Ethics approval

Ethical approval was received from the research ethics committee at Mashhad University of Medical Sciences (Code: IR.MUMS.fm.REC.1395.64).

## Results

In total 513 participants (240 males and 273 females) took part in the study, of whom 169 (74.1%) male and 220 (80.6%) female cases had a degree of hepatic steatosis. The mean age, weight, and BMI were 37.04 ± 15.44 years, 77.26 ± 17.31 kg, and 28.15 ± 4.89 kg m^2^, respectively. Overall demographic characteristics and biochemical measures are presented in Table 1. Significant differences were found in most anthropometric variables between male and female participants (see Tables 2 and 3).

**Table 1:** Demographic information of study participants.

Variable	N	Percentage (%)
Gender		
Female	270	52.6
Male	238	46.4
Diabetes		
Non-diabetic	442	85.7
Diabetic	66	13.3

	Mean ± SD	Range
Age (years)	37.04 ± 15.44	9–74
Height (cm)	165.44 ± 11.18	132–189
Weight (kg)	77.26 ± 17.31	27–141
BMI (kg/m^2^)	28.15 ± 4.89	15–52
Triglyceride (mg/dL)	130.44 ± 77.16	17–563
Total cholesterol (mg/dL)	174.99 ± 42.29	3–301
LDL-cholesterol (mg/dL)	101.28 ± 32.47	48–191
HDL-cholesterol (mg/dL)	43.61 ± 10.19	25–86
Haemoglobin (g/dL)	14.00 ± 1.84	10–24
AST (U/L)	29.98 ± 23.51	6–308
ALT (U/L)	38.28 ± 35.45	5–325
GGT (U/L)	28.29 ± 27.58	6–211
FBS (mg/dL)	96.31 ± 26.46	50–327
HbA1c (%)	5.50 ± 1.80	3–25

*SD* standard deviation, *cm* centimetres, *kg* kilograms, *BMI* body mass index, *kg/m*^*2*^ kilograms per metres squared, *mg/dL* milligrams per decilitre, *g/dL* grams per decilitre, *U/L* units per litre, *AST* aspartate aminotransferase, *ALT* alanine transaminase, *GGT* gamma-glutamyl transferase, *FBS* fasting blood glucose, *HbA1c* glycosylated haemoglobin.

**Table 2:** A comparison of the anthropometric variables across different stage of the hepatic steatosis in male and female participants.

Variables	Gender	Hepatic Steatosis				P-value
		Grade 0	Grade Ι	Grade ΙΙ	Grade ΙΙΙ	
Number	F	53	39	55	129	–
	M	81	38	48	77	–
Age, years	F	31.13 ± 15.79^#^	34.64 ± 16.67	42.02 ± 15.37^#^	41.79 ± 15.88^#^	0.001
	M	27.54 ± 12.32	35.51 ± 19.29	39.33 ± 13.91^#^	36.12 ± 13.68^#^	0.001
Weight; kg	F	59.90 ± 11.40	67.09 ± 11.08^#^	69.26 ± 11.24^#^	79.93 ± 14.56^#&$^	< 0.001
	M	66.71 ± 13.59	75.69 ± 13.90^#^	82.56 ± 10.39^#^	87.84 ± 16.70^#&^	< 0.001
Height; cm	F	156.84 ± 8.46	158.64 ± 8.04	155.83 ± 7.68	159.00 ± 6.94	0.206
	M	167.50 ± 13.60	167.78 ± 13.47	171.23 ± 6.68	172.15 ± 8.99	0.31
BMI; kg m^−2^	F	24.30 ± 4.00	26.53 ± 3.16	28.45 ± 3.44^#^	31.60 ± 5.21^#&$^	< 0.001
	M	23.83 ± 3.21	26.86 ± 3.21^#^	28.19 ± 3.53^#^	29.67 ± 4.03^#&^	< 0.001
Arm circumference; cm	F	27.60 ± 3.53	29.74 ± 3.14^#^	31.30 ± 2.58^#^	33.44 ± 4.23^#&$^	< 0.001
	M	28.81 ± 3.04	30.51 ± 2.70	31.90 ± 2.53^#^	33.27 ± 3.47^#&^	< 0.001
Neck circumference; cm	F	31.68 ± 2.37	32.77 ± 1.65	33.25 ± 1.89^#^	35.70 ± 2.34^#&$^	< 0.001
	M	36.09 ± 2.83	37.80 ± 2.73	38.94 ± 2.87^#^	39.56 ± 2.87^#&^	< 0.001
Chest circumference; cm	F	89.04 ± 8.79	94.30 ± 8.19^#^	98.78 ± 7.61^#^	106.18 ± 9.56^#&$^	< 0.001
	M	89.32 ± 10.24	96.78 ± 10.43^#^	101.34 ± 7.52^#^	104.26 ± 10.86^#&^	< 0.001
Waist circumference; cm	F	81.74 ± 10.85	86.69 ± 7.25	94.57 ± 7.92^#&^	101.96 ± 10.53^#&$^	< 0.001
	M	85.71 ± 9.08	96.42 ± 7.99^#^	99.83 ± 8.75^#^	102.68 ± 10.07^#&^	< 0.001
Abdomen circumference; cm	F	85.82 ± 11.11	91.77 ± 7.60^#^	98.15 ± 7.70^#^	105.33 ± 10.52^#&$^	< 0.001
	M	87.61 ± 8.88	98.50 ± 7.12^#^	101.27 ± 8.93^#^	104.15 ± 9.83^#&^	< 0.001
Hip circumference; cm	F	98.03 ± 12.80	101.87 ± 9.20	103.50 ± 8.82	106.60 ± 13.60^#^	0.002
	M	96.70 ± 8.88	101.31 ± 6.67	103.88 ± 7.21^#^	106.57 ± 8.00^#&^	< 0.001
Wrist circumference; cm	F	14.98 ± 0.94	15.63 ± 0.742	15.78 ± 1.13^#^	16.30 ± 1.44^#^	< 0.001
	M	16.71 ± 0.87	17.44 ± 1.11^#^	17.66 ± 0.85^#^	17.92 ± 1.10^#^	< 0.001
Subscapular skinfold; mm	F	15.40 ± 5.40	19.05 ± 5.19	21.29 ± 4.81^#^	25.33 ± 9.10^#&^	< 0.001
	M	14.01 ± 5.32	19.58 ± 7.06^#^	19.56 ± 9.05^#^	22.93 ± 8.56^#^	< 0.001
Biceps skinfold; mm	F	8.02 ± 3.29	9.47 ± 3.38	9.94 ± 2.48	12.76 ± 5.17^#&$^	< 0.001
	M	6.69 ± 3.65	8.83 ± 3.80	9.01 ± 4.14^#^	9.34 ± 3.15^#^	0.001
Triceps skinfold; mm	F	11.22 ± 3.44	14.35 ± 6.19^#^	14.02 ± 3.75	16.33 ± 6.49^#^	< 0.001
	M	9.02 ± 3.71	11.16 ± 5.05	10.40 ± 4.34	11.48 ± 3.98^#^	0.019
Suprailiac skinfold; mm	F	14.82 ± 5.27	17.59 ± 6.08	19.26 ± 6.06^#^	21.30 ± 7.54^#^	< 0.001
	M	13.98 ± 5.96	18.98 ± 7.35^#^	16.62 ± 7.41	19.42 ± 7.99^#^	0.001

Abbreviation. BMI: body mass index.

Data are presented as means ± standard deviations.

P values were obtained from analysis of variance (ANOVA), followed by Tukey’s post hoc test.

^#^Significant difference (P < 0.05) compared with grade 0; ^&^Significant difference (P < 0.05) compared with grade 1; ^$^Significant difference (P < 0.05) compared with grade 2.

**Table 3:** A comparison of the anthropometric variables across different stage of the hepatic fibrosis in male and female participants.

Variables	Gender	Hepatic fibrosis				P-value
		Grade 0	Grade Ι	Grade ΙΙ	Grade ΙΙΙ–IV	
Number	F	137	82	37	17	–
	M	156	59	14	15	–
Age, years	F	34.78 ± 16.36	38.11 ± 17.09	49.33 ± 10.64^#^	50.12 ± 8.09^#^	0.005
	M	33.05 ± 14.87	36.42 ± 14.47	36.02 ± 15.02	42.15 ± 15.30	0.119
Weight; kg	F	65.97 ± 12.73	72.39 ± 14.79^#^	81.56 ± 22.85^#^	81.77 ± 12.44^#^	< 0.001
	M	76.25 ± 14.83	83.92 ± 15.62^#^	90.08 ± 20.44^#^	90.37 ± 18.95^#^	< 0.001
Height; cm	F	157.78 ± 8.53	157.48 ± 6.70	157.55 ± 3.84	156.12 ± 6.49	0.9150
	M	169.34 ± 11.82	171.77 ± 8.39	177.08 ± 11.01	171.46 ± 9.09	0.445
BMI; kg m^−2^	F	26.41 ± 4.21	29.07 ± 4.99^#^	32.91 ± 9.21^#^	33.46 ± 4.13^#^	< 0.001
	M	26.55 ± 3.66	28.36 ± 4.41^#^	31.28 ± 4.08^#&^	30.43 ± 4.36^#^	< 0.001
Arm circumference; cm	F	29.60 ± 3.82	31.66 ± 4.24^#^	33.05 ± 7.32	33.25 ± 3.32	0.001
	M	30.83 ± 3.10	32.34 ± 3.49^#^	33.52 ± 4.23^#^	33.53 ± 4.20^#^	< 0.001
Neck circumference; cm	F	32.93 ± 2.27	33.89 ± 3.30	35.55 ± 3.08^#^	35.00 ± 3.33	0.003
	M	37.57 ± 2.84	38.98 ± 2.78^#^	40.28 ± 2.88^#^	41.15 ± 4.35^#^	< 0.001
Chest circumference; cm	F	94.30 ± 9.73	99.46 ± 10.27^#^	110.88 ± 16.56^#&^	108.18 ± 9.60^#^	< 0.001
	M	96.83 ± 11.55	100.68 ± 11.14	107.04 ± 8.37^#^	106.88 ± 10.37^#^	< 0.001
Waist circumference; cm	F	87.93 ± 11.06	94.54 ± 12.68^#^	104.83 ± 17.32^#^	105.37 ± 11.66^#^	< 0.001
	M	94.32 ± 9.73	99.82 ± 11.81^#^	106.64 ± 9.77^#&^	104.23 ± 10.25^#^	< 0.001
Abdomen circumference; cm	F	91.79 ± 10.98	99.01 ± 12.68^#^	108.27 ± 16.02^#^	108.37 ± 10.72^#^	< 0.001
	M	96.00 ± 9.25	101.15 ± 11.56^#^	108.52 ± 9.23^#&^	106.64 ± 10.55^#^	< 0.001
Hip circumference; cm	F	101.34 ± 11.28	103.33 ± 12.13	108.11 ± 17.07	104.62 ± 20.85	0.346
	M	101.22 ± 8.25	104.64 ± 8.24^#^	108.64 ± 8.76^#^	106.15 ± 8.87	< 0.001
Wrist circumference; cm	F	15.49 ± 1.15	15.71 ± 1.22	16.71 ± 1.97	16.00 ± 0.70	0.052
	M	17.44 ± 1.15	17.61 ± 0.97	18.12 ± 1.22	17..36 ± 1.31	0.075
Subscapular skinfold; mm	F	18.66 ± 6.34	21.34 ± 8.21	28.86 ± 15.23^&^	23.96 ± 5.39	0.001
	M	19.10 ± 7.03	19.56 ± 9.10	26.77 ± 10.79^#&^	22.00 ± 7.69	0.002
Biceps skinfold; mm	F	9.38 ± 3.27	10.88 ± 5.31	14.03 ± 8.77^#^	9.92 ± 2.94	0.013
	M	8.71 ± 3.80	8.60 ± 3.48	8.99 ± 3.76	8.54 ± 3.37	0.979
Triceps skinfold; mm	F	13.23 ± 4.85	13.97 ± 4.62	18.40 ± 13.00	15.44 ± 3.92	0.76
	M	10.70 ± 4.38	10.23 ± 3.77	12.95 ± 4.96	10.66 ± 3.16	0.081
Suprailiac skinfold; mm	F	17.15 ± 5.85	18.88 ± 8.27	23.97 ± 10.18^#^	19.92 ± 4.05	0.037
	M	17.32 ± 6.57	17.05 ± 7.44	23.50 ± 11.31^#&^	15.77 ± 6.70	0.004

*BMI* body mass index.

Data are presented as means ± standard deviations.

P values were obtained from analysis of variance (ANOVA), followed by Tukey’s post hoc test.

^#^Significant difference (P < 0.05) compared with grade 0; ^&^Significant difference (P < 0.05) compared with grade 1; ^$^Significant difference (P < 0.05) compared with grade 2.

### Machine learning results

**Figure 1:**
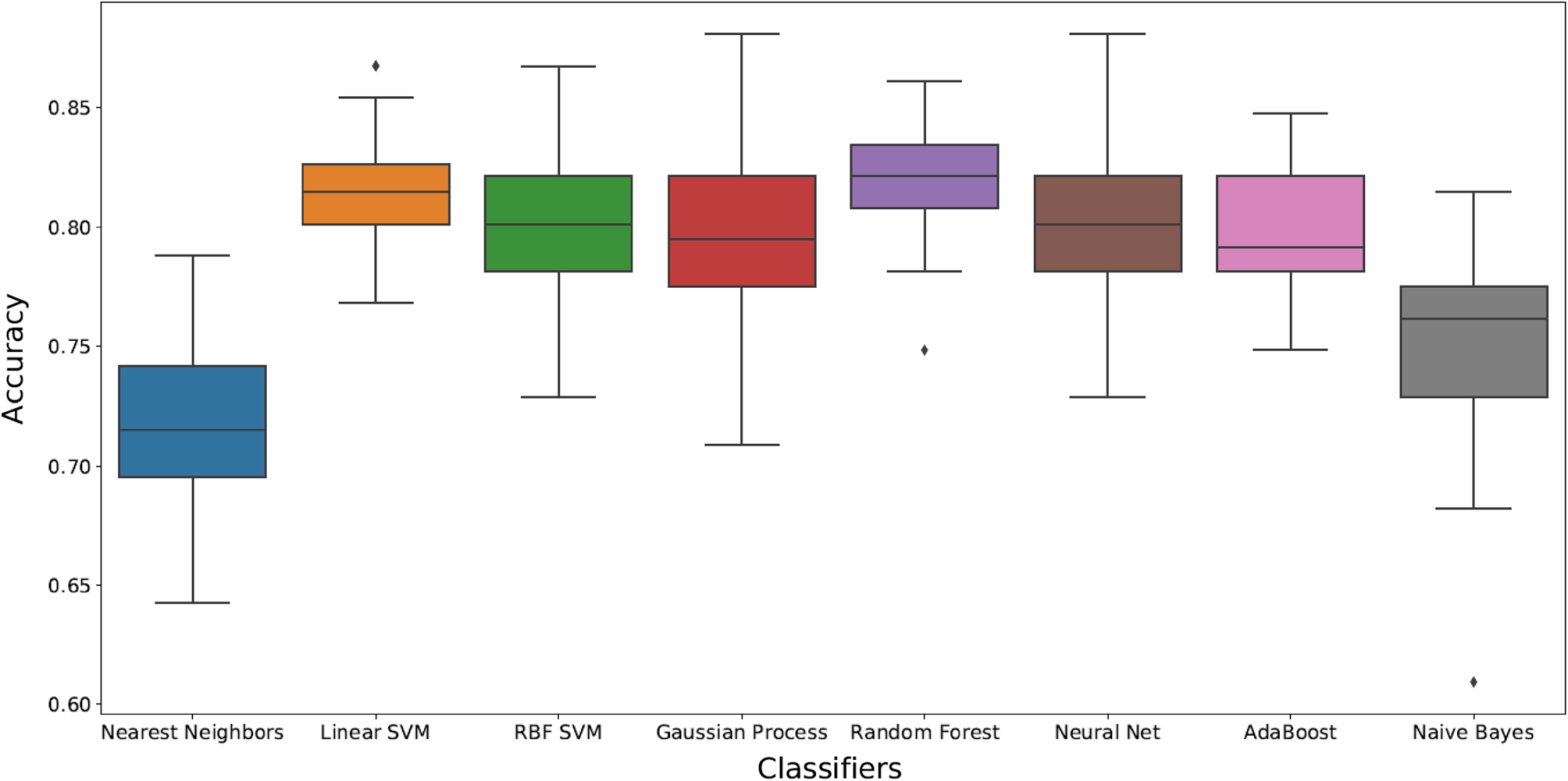
Box plots showing different classification methods applied to the dataset for presence of fatty liver. Box plots are generated by performing 50 individual runs for each classifier. This will assure that the achieved results are reliable.

**Figure 2:**
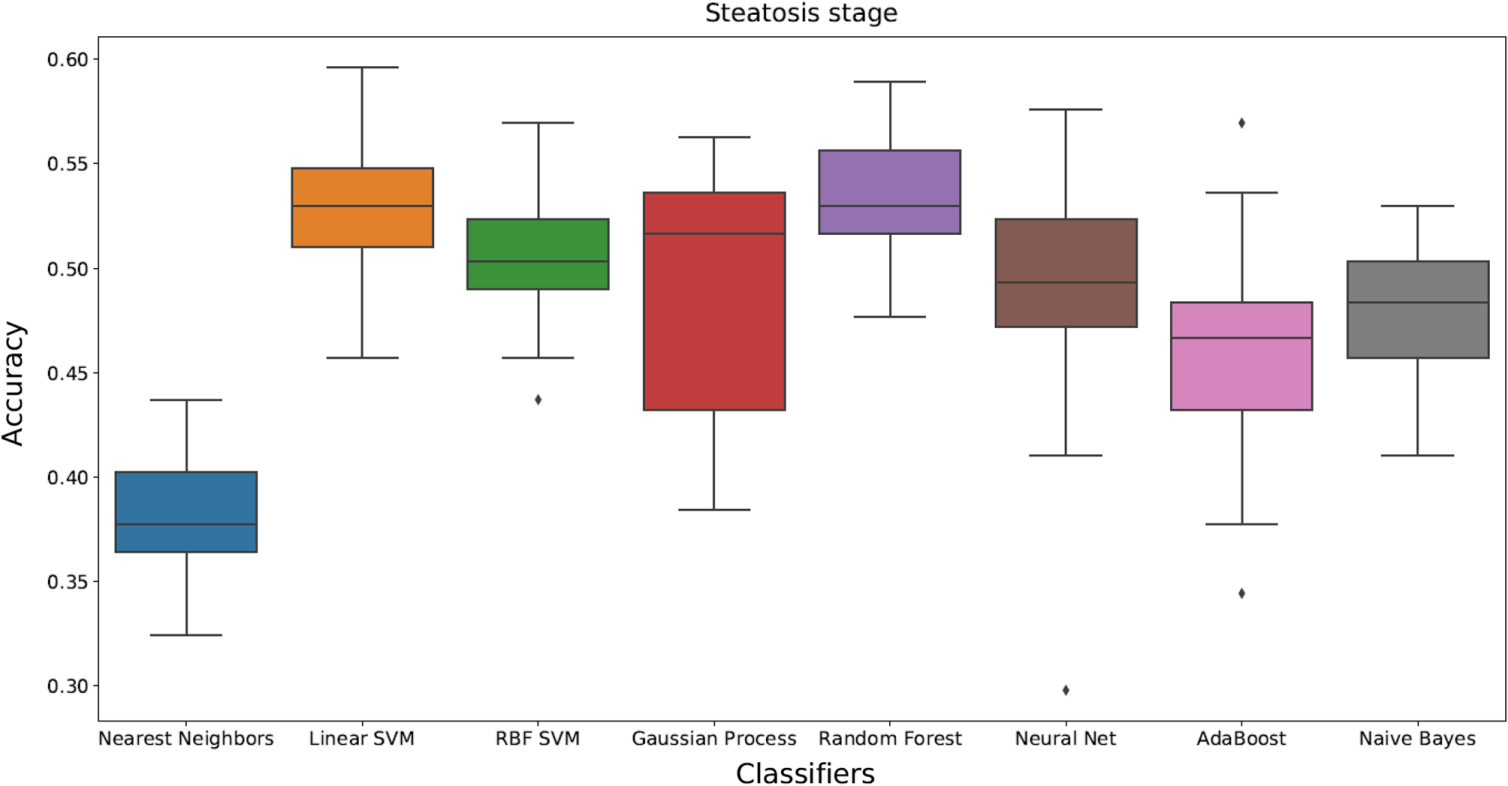
Box plots showing different classification methods applied to the dataset for stages of steatosis. Box plots are generated by performing 50 individual runs for each classifier. This will assure that the achieved results are reliable.

**Figure 3:**
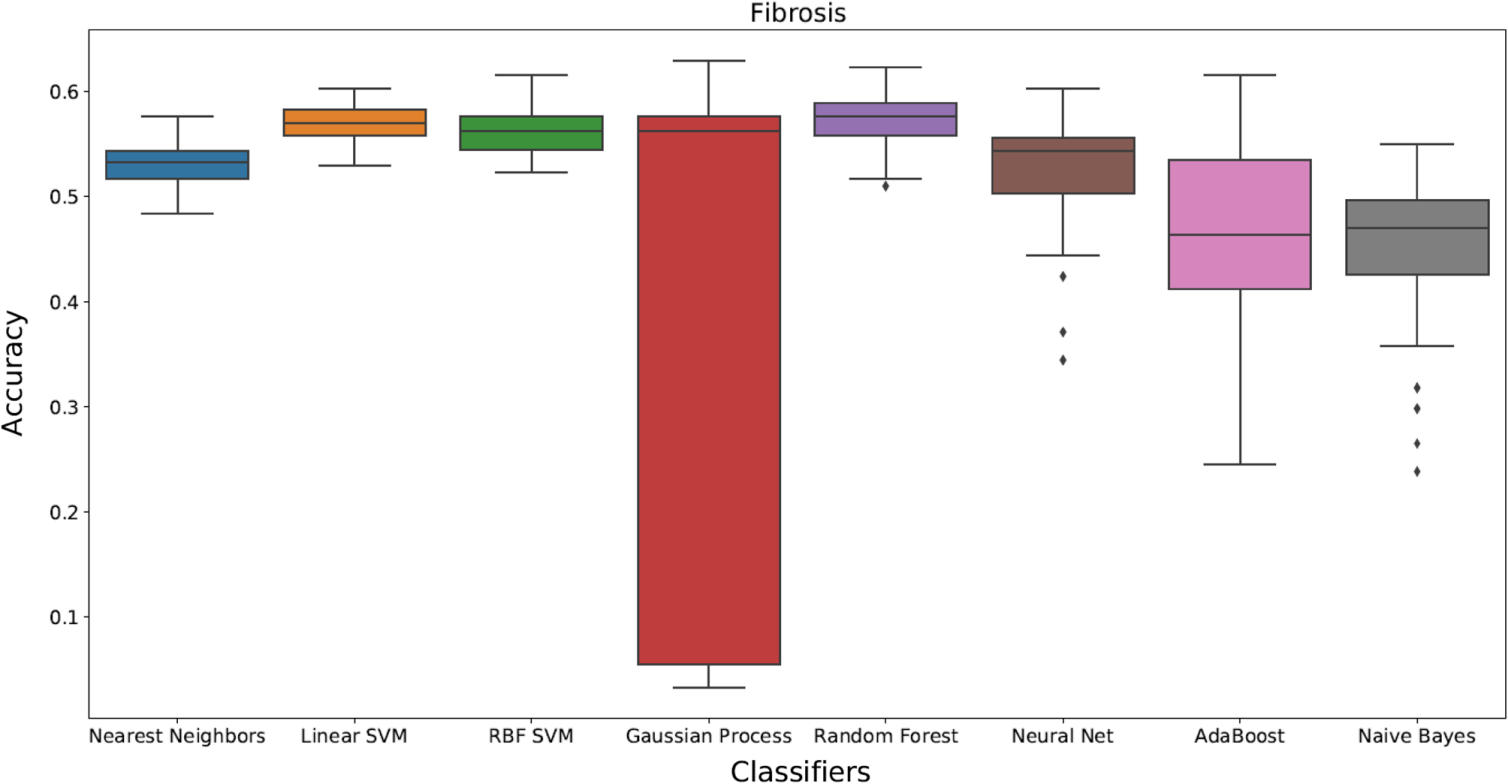
Box plots showing different classification methods applied to the dataset for stages of fibrosis. Box plots are generated by performing 50 individual runs for each classifier. This will assure that the achieved results are reliable.

Figures 1, 2, 3 present box plots for each classification method applied to three outcomes. Random Forest (RF) method generated the most accurate ML model for fatty liver (presence of any stage), steatosis stage and fibrosis stage. Average accuracy and AUC values resulted from RF were 0.82 and 0.84 for fatty liver, 0.52 and 0.69 for steatosis stages, 0.57 and 0.58 for fibrosis stages, respectively. Average accuracy and AUC are presented in the Supplemental file (Model Iterations) for all conditions. Moreover, sensitivity, specificity, true positive and true negative measures were presented for fatty liver disease.

**Figure 4:**
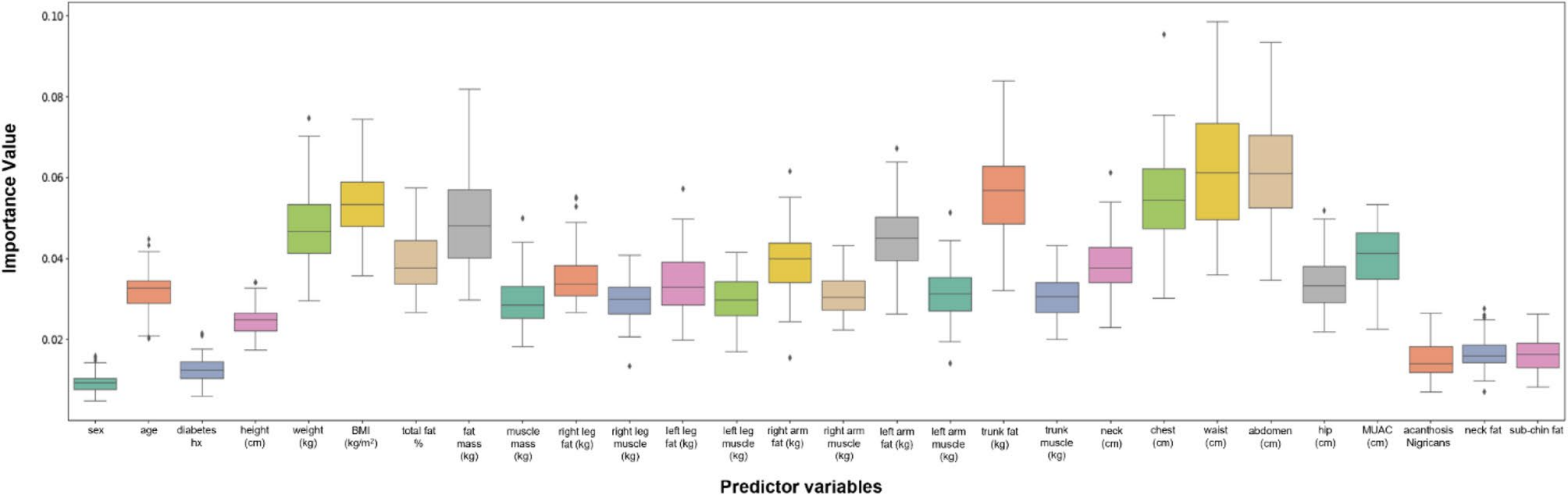
Box plots showing relative feature importance for presence of fatty liver. *hx* history, *cm* centimeter, *kg* kilograms, *BMI* body mass index, *MUAC* mid-upper arm circumference.

**Figure 5:**
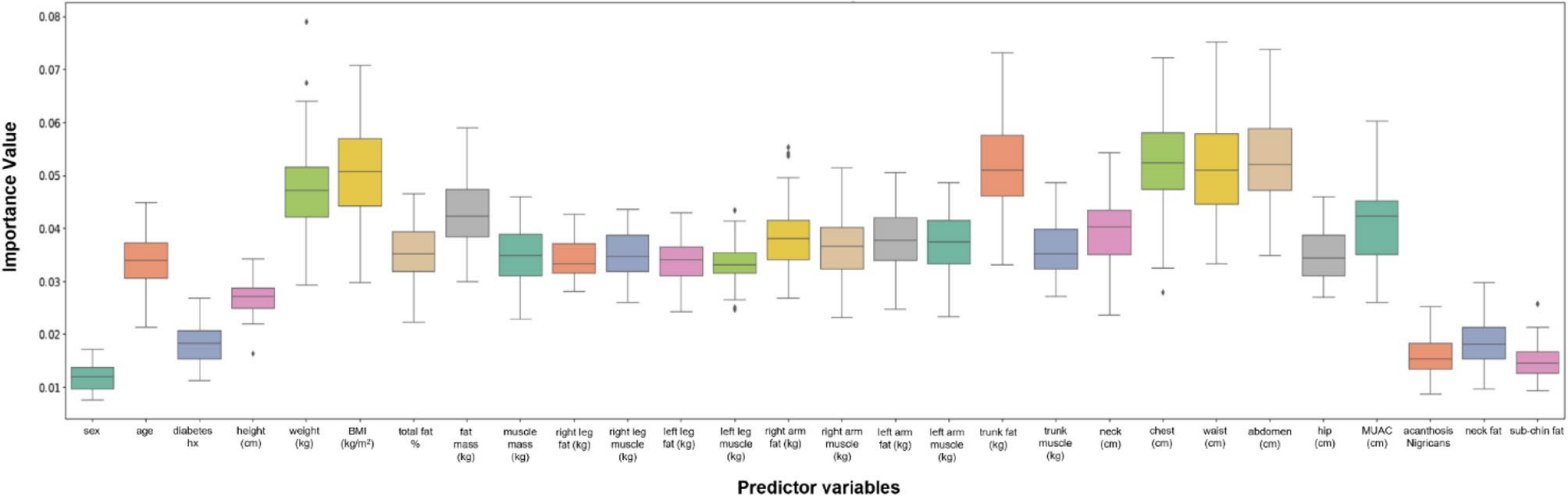
Box plots showing relative feature importance for stages of steatosis. *hx* history, *cm* centimeter, *kg* kilograms, *BMI* body mass index, *MUAC* mid-upper arm circumference.

**Figure 6:**
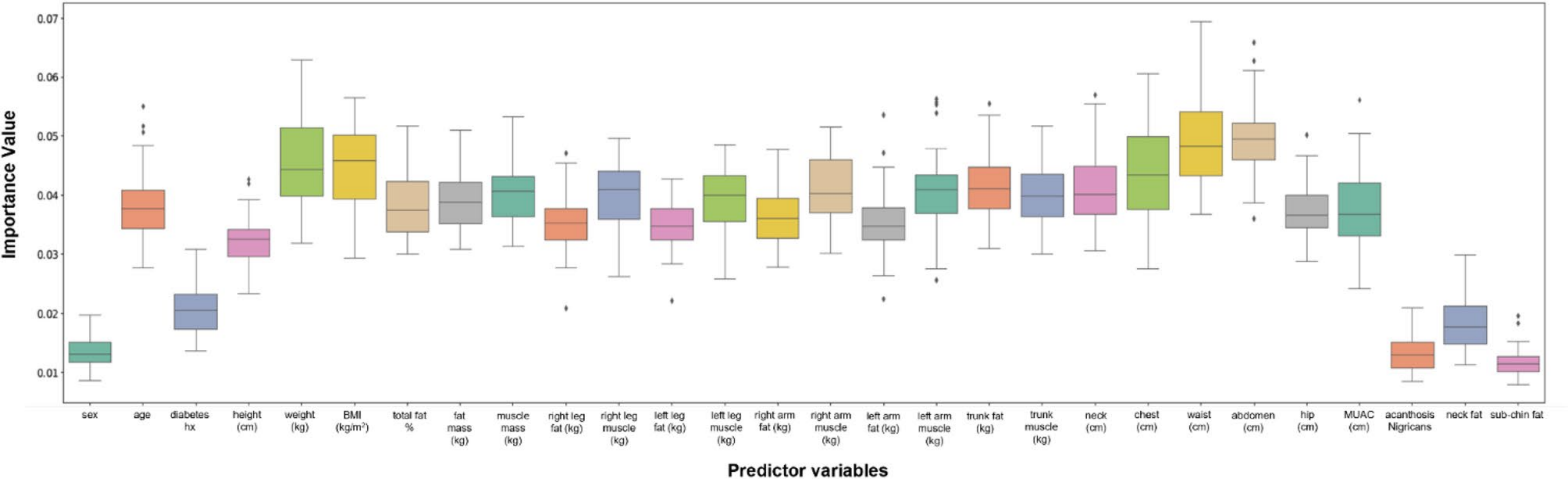
Box plots showing relative feature importance for stages of fibrosis. *hx* history, *cm* centimeter, *kg* kilograms, *BMI* body mass index, *MUAC* mid-upper arm circumference.

Feature variables with the highest predictability for fatty liver were abdomen circumference (IV; average importance value = 0.061), waist circumference (IV = 0.061), chest circumference (IV = 0.054), trunk fat (IV = 0.056) and BMI (IV = 0.053); for steatosis, the stage was abdominal circumference (IV = 0.053), waist circumference (IV = 0.052), chest circumference (IV = 0.052), trunk fat (IV = 0.051) and BMI (IV = 0.050); and for fibrosis were abdominal circumference (IV = 0.049), waist circumference (IV = 0.049), chest circumference (IV = 0.043), BMI (IV = 0.045) and weight (IV = 0.045). See Figs. 4, 5, 6 and Tables 4, 5, 6.

**Table 4:** Variable importance from the random forest method for fatty liver (presence of any stage).

Variable	Importance value
Gender	0.009424
Age	0.032057
Diabetes history	0.012520
Height	0.024799
Weight	0.047842
BMI (kg/m^2^)	0.053846
Total fat (%)	0.038906
Fat mass (kg)	0.049188
Muscle mass(kg)	0.029346
Right leg fat (kg)	0.035543
Right leg muscle (kg)	0.029538
Left leg fat (kg)	0.034062
Left leg muscle (kg)	0.030156
Right arm fat (kg)	0.039354
Right arm muscle (kg)	0.031292
Left arm fat (kg)	0.045013
Left arm muscle (kg)	0.031422
Trunk fat (kg)	0.056131
Trunk muscle (kg)	0.030482
Neck circumference (cm)	0.038175
Chest circumference (cm)	0.054649
Waist circumference (cm)	0.061709
Abdominal circumference (cm)	0.061626
Hip circumference (cm)	0.034063
Mid upper arm circumference (cm)	0.041080
Acanthosis nigricans	0.015046
Neck fat	0.016576
Sub-chin fat	0.016157

**Table 5:** Variable Importance from the random forest method for steatosis stage.

Variable	Importance value
Gender	0.011916
Age	0.033425
Diabetes history	0.018186
Height	0.026744
Weight	0.04809
BMI (kg/m^2^)	0.050049
Total fat (%)	0.035433
Fat mass (kg)	0.043273
Muscle mass(kg)	0.034973
Right leg fat (kg)	0.034236
Right leg muscle (kg)	0.035191
Left leg fat (kg)	0.033943
Left leg muscle (kg)	0.033304
Right arm fat (kg)	0.038543
Right arm muscle (kg)	0.03686
Left arm fat (kg)	0.037995
Left arm muscle (kg)	0.037145
Trunk fat (kg)	0.051388
Trunk muscle (kg)	0.035925
Neck circumference (cm)	0.039854
Chest circumference (cm)	0.05247
Waist circumference (cm)	0.052178
Abdominal circumference (cm)	0.053468
Hip circumference (cm)	0.03506
Mid upper arm circumference (cm)	0.040977
Acanthosis nigricans	0.015976
Neck fat	0.018529
Sub-chin fat	0.014871

**Table 6:** Variable importance from the random forest method for fibrosis.

Variable	Importance value
Gender	0.013453
Age	0.037938
Diabetes history	0.020626
Height	0.032533
Weight	0.045225
BMI (kg/m^2^)	0.045191
Total fat (%)	0.038413
Fat mass (kg)	0.039101
Muscle mass(kg)	0.041035
Right leg fat (kg)	0.035216
Right leg muscle (kg)	0.040116
Left leg fat (kg)	0.034831
Left leg muscle (kg)	0.039268
Right arm fat (kg)	0.036479
Right arm muscle (kg)	0.041001
Left arm fat (kg)	0.035263
Left arm muscle (kg)	0.040377
Trunk fat (kg)	0.041605
Trunk muscle (kg)	0.040320
Neck circumference (cm)	0.040930
Chest circumference (cm)	0.043684
Waist circumference (cm)	0.049580
Abdominal circumference (cm)	0.049180
Hip circumference (cm)	0.037576
Mid upper arm circumference (cm)	0.037792
Acanthosis nigricans	0.013215
Neck fat	0.018472
Sub-chin fat	0.011579

Further assessment identified gender-specific features (see Supplemental Figs. 1–6; Supplemental Tables 1–6). Important predictor factors for fatty liver disease among females were waist circumference (IV = 0.057), abdomen circumference (IV = 0.056), trunk fat (IV = 0.055), fat mass (IV = 0.052), chest circumference (IV = 0.048), and BMI (IV = 0.048) were the most important features. Among males, waist circumference (IV = 0.053), chest circumference (IV = 0.052), trunk fat (IV = 0.051), BMI (IV = 0.052), abdomen circumference (IV = 0.049) and fat mass (IV = 0.048) had the highest predictive value for fatty liver. Important predictor factors for steatosis among females were abdomen circumference (IV = 0.048), waist circumference (IV = 0.047), weight (IV = 0.046), trunk fat (IV = 0.045), fat mass (IV = 0.044), and BMI (IV = 0.043) were the most important features. Among males, waist circumference (IV = 0.051), chest circumference (IV = 0.050), abdomen circumference (IV = 0.049), trunk fat (IV = 0.048), BMI (IV = 0.048), and fat mass (IV = 0.046) had the highest predictive value for steatosis. Important predictor factors for fibrosis among females were abdomen circumference (IV = 0.048), waist circumference (IV = 0.047), BMI (IV = 0.046), trunk fat (IV = 0.045), chest circumference (IV = 0.043), and muscle mass (IV = 0.043) were the most important features. Among males, abdomen circumference (IV = 0.045), waist circumference (IV = 0.043), weight (IV = 0.043), BMI (IV = 0.043), right arm fat (IV = 0.042) and fat mass (IV = 0.042) had the highest predictive value for fibrosis.

## Discussion

This study applied ML techniques to determine the optimal body composition and anthropometric classifier of NAFLD and identify feature contribution to the prediction of the disease. RF generated the most accurate ML model to predict fatty liver presence, steatosis (stages) and fibrosis. To our knowledge, this is the first study applying ML on body composition and anthropometric data to predict NAFLD. High accuracy (82%) highlights the potential for applying ML techniques for the primary prevention and screening of NAFLD using anthropometric measurements.

Previous studies using ML techniques to predict fatty liver disease have mainly focused on biochemical measurements, with similar levels of accuracy (83.0%) using Bayesian Network^38^, (76.3%) Logistic Regression^37^, (86.4%) RF^39^ and (80%) Classification Tree techniques^36^. However, we tested the predictive value of body composition and anthropometric measurements rather than biochemical variables. Anthropometry as a lower-cost and more feasible approach can be considered a primary screening method for fatty liver disease.

Abdominal obesity is a significant risk factor leading to NAFLD^27^. Waist circumference and trunk fat have been shown to be significantly predicting the risk of NAFLD^24^. Although BMI is one of the risk factors of NAFLD^31^, it has been argued that BMI is limited compared to other anthropometric measures (e.g., waist circumference) in identifying lean NAFLD individuals^25^. In a similar vein, the findings of the present study clearly show the importance of these body composition and anthropometric measures and their relative contribution to the prediction of NAFLD.

Neck circumference reflects the amount of subcutaneous fat in the upper body, and is a reliable factor in determining central obesity^48^. A positive correlation has been shown between neck circumference and hepatic steatosis^26,28^. Neck circumference showed a positive association with other anthropometric components, such as BMI and waist and waist-to-hip circumference. In the present study, neck circumference contributed almost equally to hepatic steatosis and fibrosis.

A study by Subramanian revealed that the level of arm fat index in both males and females had a negative association with the degree and severity of NAFLD^29^. In our study, a strong and positive relationship between arm circumference and the severity of steatosis and fibrosis was detected, validated by the ML model. Rafiee et al. showed that the amount of fat in hips and legs and circumference of hip negatively associated with fatty liver and the severity of the disease. In contrast, the waist-to-hip ratio was closely associated with fatty liver. They also showed that the accuracy of this ratio in predicting NAFLD was greater than BMI and waist-to-height ratio^49^.

Most ML studies for the prediction of NAFLD have used the ultrasonography technique to diagnose fatty liver disease^36–39^. Ultrasound is a commonly used method for the diagnosis of hepatic steatosis^50^. Ultrasonography is a safe, well-tolerated, non-invasive and low-cost technique^50^; however, there are limitations associated with ultrasound use, including limited capability in detecting fatty infiltration (less than 20% steatosis), operator dependency and subjective assessment^51,52^, and ML is expected to minimise some of these. Application of ML techniques on body composition and anthropometric measures as a less time-consuming and easy to undertake method can help physicians in their clinical decision making.

The presence of liver fibrosis in patients with NAFLD is considered the strongest predictor of long-term outcome^53^. NAFLD Fibrosis Score (NFS) and Fibrosis-4 (FIB-4) have been recommended as appropriate methods for the initial assessment of fibrosis in NAFLD patients^54^. Both of these methods use a combination of variables including age, BMI and biochemical measures (i.e. aspartate aminotransferase (AST), alanine aminotransferase (ALT), platelets, etc.). Graupera et al. concluded that NFS and FIB-4 are not optimal for screening as they correlate poorly with liver stiffness^55^. In their study, waist circumference was found to be the ideal measure for fibrosis screening among high risk people from general population^55^. However, other studies found that NFS and FIB-4 have the potential to detect advanced fibrosis and the progression of fibrosis among people with NAFLD^56^. It seems that NFS and FIB-4 are more useful in the diagnosis of fibrosis in NAFLD but not for fibrosis screening among the general populations. The present study showed suboptimal accuracy (57%) in detecting fibrosis using less expensive and non-invasive factors i.e. anthropometric and body composition measures. Further studies might explore a combination of these methods including anthropometric, body composition and biochemical variables altogether.

The proposed algorithm identified in this research can be used by the health systems for several reasons. Screening of the presence or absence of NAFLD with the help of non-invasive anthropometric measurements can be achieved with simple and cheap equipment^57^. Moreover, performing the measurement task needs less specialty knowledge therefore can be implemented in several health centres (e.g., primary practice) and also remote areas. Once validated, the resulted assistive technology can serve the clinicians in the prevention of liver diseases. There are limitations of the present research that need to be addressed. A small sample size might have potentially limited the results of ML prediction. Although, the small sample size was accounted for by multiple cross-validations, which reduced potential errors. Future studies with larger sample sizes can allocate separate validation sets and evaluate the model. Moreover, even though the most common method for fatty liver diagnosis, the ultrasound technique is not the gold standard. Using liver biopsy outcomes would generate more valid results. Also, to increase the predictive accuracy of the proposed model for NAFLD prediction, future studies should include other body composition and anthropometric measures such as sagittal abdominal diameter (SAD) and peri-renal fat^58^.

## Conclusion

Present findings show that applying a ML classification model on anthropometric and body composition variables predicted the presence of fatty liver disease. ML-based decision support systems offer potential to assist physicians with screening, diagnosis and prevention of NAFLD. ML-based decision support systems could be of particular value for providing services at a population level and remote health care where there is a lack of trained specialists.

## Supplementary Information

Supplementary Information 1.

Supplementary Information 2.

## Data Availability

The datasets used and/or analyzed in the current study are available from the corresponding author upon reasonable request.

## Abbreviations

NAFLD: Non-alcoholic fatty liver disease
BMI: Body mass index
LSM: Liver stiffness measurement
ROCc: Receiver operating characteristic curve
PCA: Principal component analysis
CAP: Controlled attenuation parameter
CI: Confidence interval
ANOVA: Analysis of variance
DCL: Dorso-cervical lipohypertrophy
