# Supplementary material for: Application of machine learning in predicting non-alcoholic fatty liver disease using anthropometric and body composition indices
Source: Sci Rep. 2023 Mar 27;13:4942. doi: 10.1038/s41598-023-32129-y (PMC10043285; doi:10.1038/s41598-023-32129-y)
Supplement: Supplementary file 1 — Supplementary Information 1. [file 41598_2023_32129_MOESM1_ESM.docx]

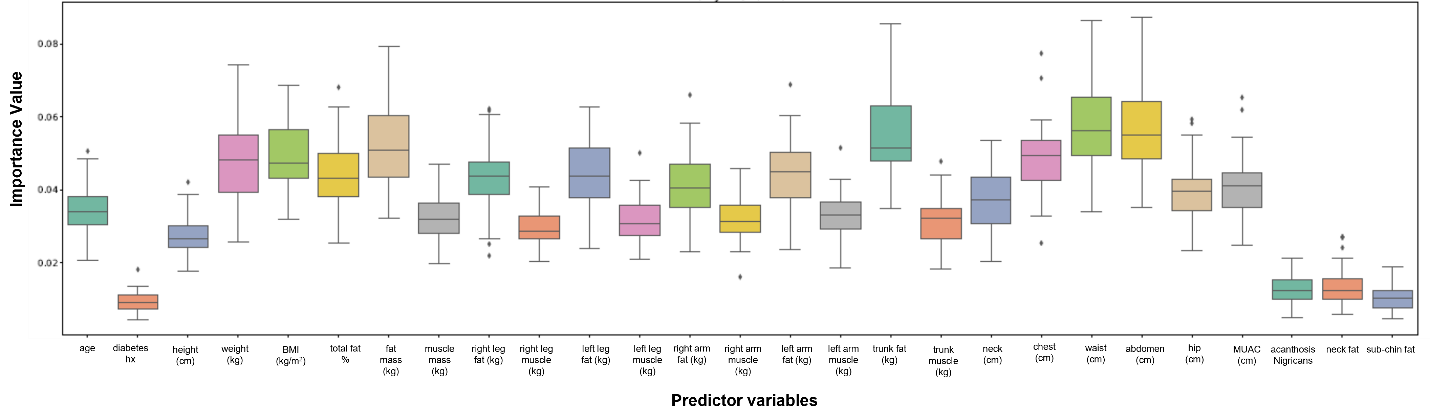


Supplemental Figure 1. Box plots showing relative feature importance for presence of fatty liver in females.

hx, history; cm, centimeter; kg, kilograms; BMI, body mass index; MUAC, mid-upper arm circumference.


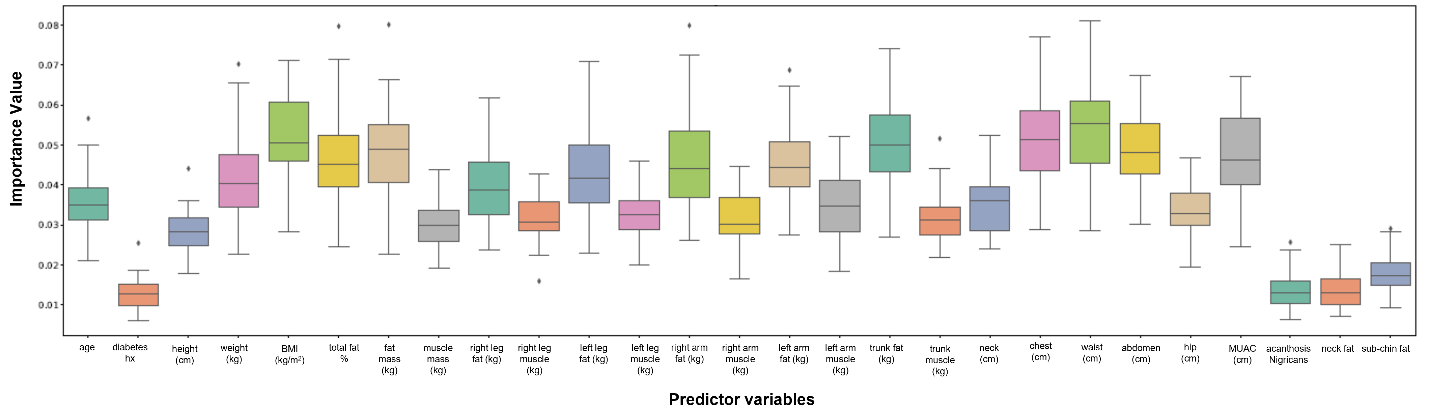


Supplemental Figure 2. Box plots showing relative feature importance for presence of fatty liver in males.

hx, history; cm, centimeter; kg, kilograms; BMI, body mass index; MUAC, mid-upper arm circumference.


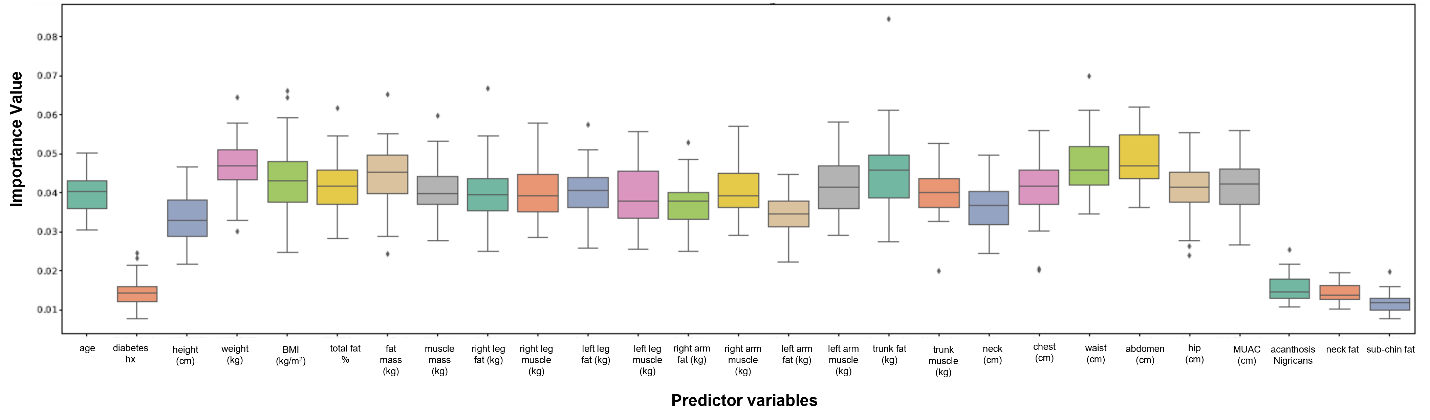


Supplemental Figure 3. Box plots showing relative feature importance for stages of steatosis in females.

hx, history; cm, centimeter; kg, kilograms; BMI, body mass index; MUAC, mid-upper arm circumference.


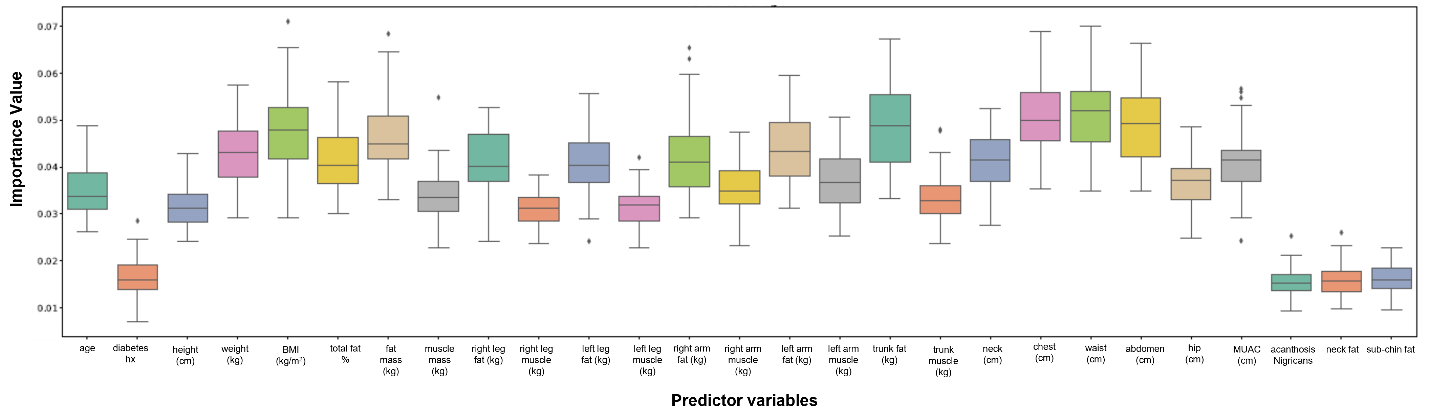


Supplemental Figure 4. Box plots showing relative feature importance for stages of steatosis in males.

hx, history; cm, centimeter; kg, kilograms; BMI, body mass index; MUAC, mid-upper arm circumference.


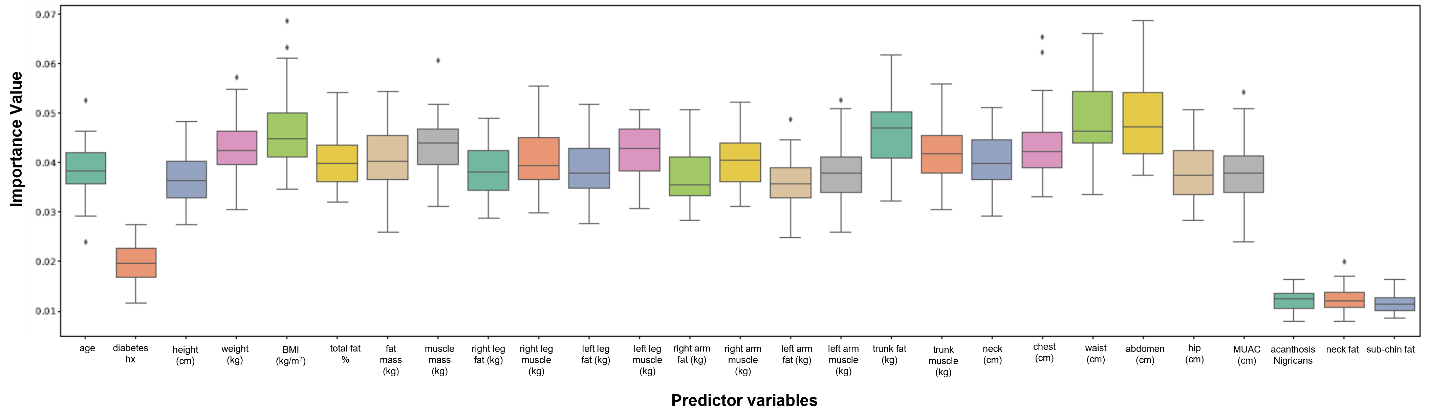


Supplemental Figure 5. Box plots showing relative feature importance for stages of fibrosis in females.

hx, history; cm, centimeter; kg, kilograms; BMI, body mass index; MUAC, mid-upper arm circumference.


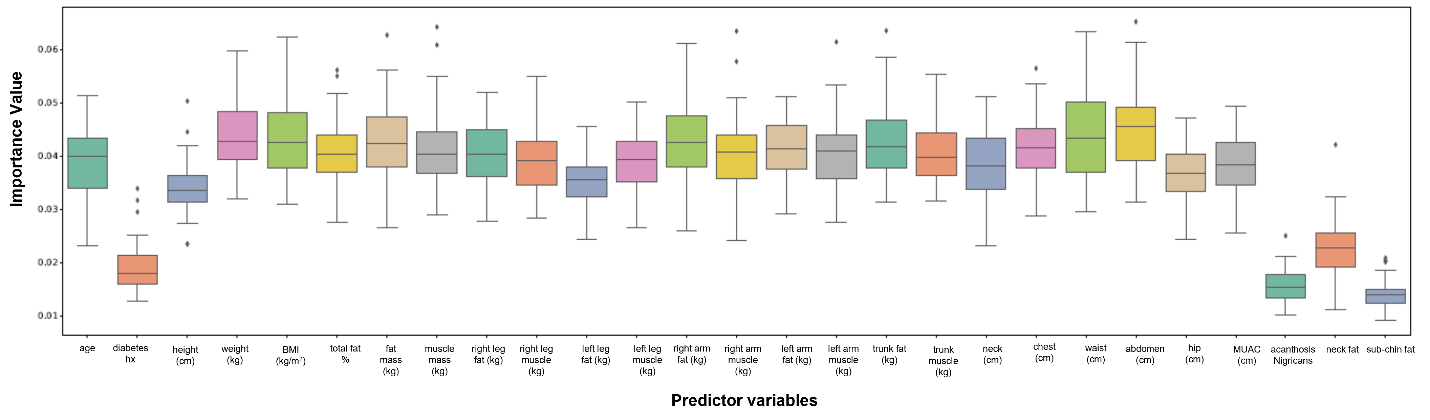


Supplemental Figure 6. Box plots showing relative feature importance for stages of fibrosis in males.

hx, history; cm, centimeter; kg, kilograms; BMI, body mass index; MUAC, mid-upper arm circumference.

| **Supplemental Table 1. Variable Importance from The Random Forest Method for Fatty Liver (Presence of Any Stage) in Females.** | |
| --- | --- |
| **Variable** | **Importance Value** |
| Age | 0.034628 |
| Diabetes History | 0.009427 |
| Height | 0.027276 |
| Weight | 0.048199 |
| BMI (kg/m2) | 0.048589 |
| Total Fat (%) | 0.044906 |
| Fat Mass (kg) | 0.052164 |
| Muscle Mass(kg) | 0.032092 |
| Right Leg Fat (kg) | 0.043792 |
| Right Leg Muscle (kg) | 0.029332 |
| Left Leg Fat (kg) | 0.043804 |
| Left Leg Muscle (kg) | 0.031668 |
| Right Arm Fat (kg) | 0.041417 |
| Right Arm Muscle (kg) | 0.031812 |
| Left Arm Fat (kg) | 0.044008 |
| Left Arm Muscle (kg) | 0.032732 |
| Trunk Fat (kg) | 0.055924 |
| Trunk Muscle (kg) | 0.031348 |
| Neck Circumference (cm) | 0.037783 |
| Chest Circumference (cm) | 0.048633 |
| Waist Circumference (cm) | 0.057876 |
| Abdominal Circumference (cm) | 0.056571 |
| Hip Circumference (cm) | 0.038917 |
| Mid Upper Arm Circumference (cm) | 0.040829 |
| Acanthosis Nigricans | 0.012650 |
| Neck Fat | 0.013504 |
| Sub-chin Fat | 0.010117 |

| **Supplemental Table 2. Variable Importance from The Random Forest Method for Fatty Liver (Presence of Any Stage) in Males.** | |
| --- | --- |
| **Variable** | **Importance Value** |
| Age | 0.035738 |
| Diabetes History | 0.012512 |
| Height | 0.028341 |
| Weight | 0.042224 |
| BMI (kg/m2) | 0.052151 |
| Total Fat (%) | 0.046328 |
| Fat Mass (kg) | 0.048605 |
| Muscle Mass(kg) | 0.030101 |
| Right Leg Fat (kg) | 0.040062 |
| Right Leg Muscle (kg) | 0.031686 |
| Left Leg Fat (kg) | 0.043051 |
| Left Leg Muscle (kg) | 0.032153 |
| Right Arm Fat (kg) | 0.045651 |
| Right Arm Muscle (kg) | 0.031581 |
| Left Arm Fat (kg) | 0.045161 |
| Left Arm Muscle (kg) | 0.035518 |
| Trunk Fat (kg) | 0.051096 |
| Trunk Muscle (kg) | 0.031575 |
| Neck Circumference (cm) | 0.035179 |
| Chest Circumference (cm) | 0.052099 |
| Waist Circumference (cm) | 0.053858 |
| Abdominal Circumference (cm) | 0.049417 |
| Hip Circumference (cm) | 0.033419 |
| Mid Upper Arm Circumference (cm) | 0.047281 |
| Acanthosis Nigricans | 0.013631 |
| Neck Fat | 0.013616 |
| Sub-chin Fat | 0.017968 |

| **Supplemental Table 3. Variable Importance from The Random Forest Method for Steatosis in Females.** | |
| --- | --- |
| **Variable** | **Importance Value** |
| Age | 0.039751 |
| Diabetes History | 0.014293 |
| Height | 0.033268 |
| Weight | 0.046651 |
| BMI (kg/m2) | 0.043300 |
| Total Fat (%) | 0.041575 |
| Fat Mass (kg) | 0.044511 |
| Muscle Mass(kg) | 0.040742 |
| Right Leg Fat (kg) | 0.040233 |
| Right Leg Muscle (kg) | 0.040045 |
| Left Leg Fat (kg) | 0.039584 |
| Left Leg Muscle (kg) | 0.039301 |
| Right Arm Fat (kg) | 0.037333 |
| Right Arm Muscle (kg) | 0.040767 |
| Left Arm Fat (kg) | 0.034641 |
| Left Arm Muscle (kg) | 0.041709 |
| Trunk Fat (kg) | 0.045336 |
| Trunk Muscle (kg) | 0.039824 |
| Neck Circumference (cm) | 0.036295 |
| Chest Circumference (cm) | 0.041153 |
| Waist Circumference (cm) | 0.047099 |
| Abdominal Circumference (cm) | 0.048332 |
| Hip Circumference (cm) | 0.040996 |
| Mid Upper Arm Circumference (cm) | 0.041878 |
| Acanthosis Nigricans | 0.015398 |
| Neck Fat | 0.014403 |
| Sub-chin Fat | 0.011583 |

| **Supplemental Table 4. Variable Importance from The Random Forest Method for Steatosis in Males.** | |
| --- | --- |
| **Variable** | **Importance Value** |
| Age | 0.034960 |
| Diabetes History | 0.016353 |
| Height | 0.031464 |
| Weight | 0.042667 |
| BMI (kg/m2) | 0.048233 |
| Total Fat (%) | 0.041570 |
| Fat Mass (kg) | 0.046288 |
| Muscle Mass(kg) | 0.034046 |
| Right Leg Fat (kg) | 0.040738 |
| Right Leg Muscle (kg) | 0.031170 |
| Left Leg Fat (kg) | 0.040802 |
| Left Leg Muscle (kg) | 0.031729 |
| Right Arm Fat (kg) | 0.042121 |
| Right Arm Muscle (kg) | 0.035777 |
| Left Arm Fat (kg) | 0.044034 |
| Left Arm Muscle (kg) | 0.037246 |
| Trunk Fat (kg) | 0.048987 |
| Trunk Muscle (kg) | 0.033405 |
| Neck Circumference (cm) | 0.041464 |
| Chest Circumference (cm) | 0.050574 |
| Waist Circumference (cm) | 0.051518 |
| Abdominal Circumference (cm) | 0.049248 |
| Hip Circumference (cm) | 0.036631 |
| Mid Upper Arm Circumference (cm) | 0.041083 |
| Acanthosis Nigricans | 0.015646 |
| Neck Fat | 0.015985 |
| Sub-chin Fat | 0.016262 |

| **Supplemental Table 5. Variable Importance from The Random Forest Method for Fibrosis in Females.** | |
| --- | --- |
| **Variable** | **Importance Value** |
| Age | 0.038383 |
| Diabetes History | 0.019977 |
| Height | 0.036515 |
| Weight | 0.042881 |
| BMI (kg/m2) | 0.046026 |
| Total Fat (%) | 0.040227 |
| Fat Mass (kg) | 0.040830 |
| Muscle Mass(kg) | 0.043101 |
| Right Leg Fat (kg) | 0.038594 |
| Right Leg Muscle (kg) | 0.040777 |
| Left Leg Fat (kg) | 0.038831 |
| Left Leg Muscle (kg) | 0.042164 |
| Right Arm Fat (kg) | 0.036841 |
| Right Arm Muscle (kg) | 0.040268 |
| Left Arm Fat (kg) | 0.035891 |
| Left Arm Muscle (kg) | 0.038002 |
| Trunk Fat (kg) | 0.045943 |
| Trunk Muscle (kg) | 0.041706 |
| Neck Circumference (cm) | 0.040531 |
| Chest Circumference (cm) | 0.043191 |
| Waist Circumference (cm) | 0.047978 |
| Abdominal Circumference (cm) | 0.048512 |
| Hip Circumference (cm) | 0.038257 |
| Mid Upper Arm Circumference (cm) | 0.038311 |
| Acanthosis Nigricans | 0.012326 |
| Neck Fat | 0.012380 |
| Sub-chin Fat | 0.011558 |

| **Supplemental Table 6. Variable Importance from The Random Forest Method for Fibrosis in Males.** | |
| --- | --- |
| **Variable** | **Importance Value** |
| Age | 0.039315 |
| Diabetes History | 0.018903 |
| Height | 0.034160 |
| Weight | 0.043558 |
| BMI (kg/m2) | 0.043370 |
| Total Fat (%) | 0.040628 |
| Fat Mass (kg) | 0.042548 |
| Muscle Mass(kg) | 0.041416 |
| Right Leg Fat (kg) | 0.039990 |
| Right Leg Muscle (kg) | 0.039226 |
| Left Leg Fat (kg) | 0.035132 |
| Left Leg Muscle (kg) | 0.038777 |
| Right Arm Fat (kg) | 0.042628 |
| Right Arm Muscle (kg) | 0.040456 |
| Left Arm Fat (kg) | 0.040802 |
| Left Arm Muscle (kg) | 0.040869 |
| Trunk Fat (kg) | 0.042598 |
| Trunk Muscle (kg) | 0.040476 |
| Neck Circumference (cm) | 0.037792 |
| Chest Circumference (cm) | 0.041495 |
| Waist Circumference (cm) | 0.043666 |
| Abdominal Circumference (cm) | 0.045073 |
| Hip Circumference (cm) | 0.036649 |
| Mid Upper Arm Circumference (cm) | 0.038163 |
| Acanthosis Nigricans | 0.015607 |
| Neck Fat | 0.022678 |
| Sub-chin Fat | 0.014024 |
