# Supplementary material for: Application of machine learning in predicting non-alcoholic fatty liver disease using anthropometric and body composition indices
Source: Sci Rep. 2023 Mar 27;13:4942. doi: 10.1038/s41598-023-32129-y (PMC10043285; doi:10.1038/s41598-023-32129-y)
Supplement: Supplementary file 2 — Supplementary Information 2. [file 41598_2023_32129_MOESM2_ESM.docx]

| Table 1. Fifty-times iteration of models for presence or absence of Fatty Liver. | | | | | | | |
| --- | --- | --- | --- | --- | --- | --- | --- |
| Column1 | **ClfName** | **Accuracy** | **AUC** | **Sensitivity** | **Specificity** | **True Positive** | **True Negative** |
| 0 | Nearest Neighbors | 0.7218543 | 0.68109 | 0.732 | 0.677 | 82.020 | 26.400 |
| 1 | Nearest Neighbors | 0.7152318 | 0.73054 |  |  |  |  |
| 2 | Nearest Neighbors | 0.6953642 | 0.687157 |  |  |  |  |
| 3 | Nearest Neighbors | 0.7218543 | 0.723558 |  |  |  |  |
| 4 | Nearest Neighbors | 0.7549669 | 0.816621 |  |  |  |  |
| 5 | Nearest Neighbors | 0.6953642 | 0.740385 |  |  |  |  |
| 6 | Nearest Neighbors | 0.6490066 | 0.640568 |  |  |  |  |
| 7 | Nearest Neighbors | 0.7152318 | 0.744277 |  |  |  |  |
| 8 | Nearest Neighbors | 0.7218543 | 0.713599 |  |  |  |  |
| 9 | Nearest Neighbors | 0.7483444 | 0.756296 |  |  |  |  |
| 10 | Nearest Neighbors | 0.7350993 | 0.728594 |  |  |  |  |
| 11 | Nearest Neighbors | 0.6754967 | 0.656593 |  |  |  |  |
| 12 | Nearest Neighbors | 0.6821192 | 0.720467 |  |  |  |  |
| 13 | Nearest Neighbors | 0.7350993 | 0.755838 |  |  |  |  |
| 14 | Nearest Neighbors | 0.7682119 | 0.749542 |  |  |  |  |
| 15 | Nearest Neighbors | 0.7483444 | 0.775069 |  |  |  |  |
| 16 | Nearest Neighbors | 0.7019868 | 0.721612 |  |  |  |  |
| 17 | Nearest Neighbors | 0.7086093 | 0.752289 |  |  |  |  |
| 18 | Nearest Neighbors | 0.7152318 | 0.673535 |  |  |  |  |
| 19 | Nearest Neighbors | 0.7284768 | 0.690018 |  |  |  |  |
| 20 | Nearest Neighbors | 0.7284768 | 0.732601 |  |  |  |  |
| 21 | Nearest Neighbors | 0.7152318 | 0.761905 |  |  |  |  |
| 22 | Nearest Neighbors | 0.7019868 | 0.694139 |  |  |  |  |
| 23 | Nearest Neighbors | 0.7748344 | 0.796932 |  |  |  |  |
| 24 | Nearest Neighbors | 0.7615894 | 0.76511 |  |  |  |  |
| 25 | Nearest Neighbors | 0.7417219 | 0.731799 |  |  |  |  |
| 26 | Nearest Neighbors | 0.6556291 | 0.697459 |  |  |  |  |
| 27 | Nearest Neighbors | 0.7218543 | 0.737866 |  |  |  |  |
| 28 | Nearest Neighbors | 0.7218543 | 0.697459 |  |  |  |  |
| 29 | Nearest Neighbors | 0.6821192 | 0.751374 |  |  |  |  |
| 30 | Nearest Neighbors | 0.7350993 | 0.750343 |  |  |  |  |
| 31 | Nearest Neighbors | 0.7218543 | 0.731799 |  |  |  |  |
| 32 | Nearest Neighbors | 0.781457 | 0.802198 |  |  |  |  |
| 33 | Nearest Neighbors | 0.7152318 | 0.752633 |  |  |  |  |
| 34 | Nearest Neighbors | 0.7284768 | 0.713828 |  |  |  |  |
| 35 | Nearest Neighbors | 0.6688742 | 0.695971 |  |  |  |  |
| 36 | Nearest Neighbors | 0.7284768 | 0.741758 |  |  |  |  |
| 37 | Nearest Neighbors | 0.7483444 | 0.768201 |  |  |  |  |
| 38 | Nearest Neighbors | 0.7218543 | 0.748397 |  |  |  |  |
| 39 | Nearest Neighbors | 0.6754967 | 0.682349 |  |  |  |  |
| 40 | Nearest Neighbors | 0.7019868 | 0.742445 |  |  |  |  |
| 41 | Nearest Neighbors | 0.6225166 | 0.66701 |  |  |  |  |
| 42 | Nearest Neighbors | 0.7086093 | 0.730082 |  |  |  |  |
| 43 | Nearest Neighbors | 0.7350993 | 0.731914 |  |  |  |  |
| 44 | Nearest Neighbors | 0.7615894 | 0.765339 |  |  |  |  |
| 45 | Nearest Neighbors | 0.7748344 | 0.748397 |  |  |  |  |
| 46 | Nearest Neighbors | 0.7019868 | 0.659341 |  |  |  |  |
| 47 | Nearest Neighbors | 0.7019868 | 0.699405 |  |  |  |  |
| 48 | Nearest Neighbors | 0.6754967 | 0.679029 |  |  |  |  |
| 49 | Nearest Neighbors | 0.7019868 | 0.686813 |  |  |  |  |
| 50 | Linear SVM | 0.8145695 | 0.859432 | 0.937 | 0.485 | 104.980 | 18.900 |
| 51 | Linear SVM | 0.7880795 | 0.819368 |  |  |  |  |
| 52 | Linear SVM | 0.8344371 | 0.876374 |  |  |  |  |
| 53 | Linear SVM | 0.8410596 | 0.867674 |  |  |  |  |
| 54 | Linear SVM | 0.807947 | 0.789377 |  |  |  |  |
| 55 | Linear SVM | 0.8211921 | 0.845925 |  |  |  |  |
| 56 | Linear SVM | 0.8278146 | 0.837225 |  |  |  |  |
| 57 | Linear SVM | 0.8013245 | 0.836538 |  |  |  |  |
| 58 | Linear SVM | 0.807947 | 0.827381 |  |  |  |  |
| 59 | Linear SVM | 0.8145695 | 0.834936 |  |  |  |  |
| 60 | Linear SVM | 0.8344371 | 0.887592 |  |  |  |  |
| 61 | Linear SVM | 0.781457 | 0.797161 |  |  |  |  |
| 62 | Linear SVM | 0.8211921 | 0.874313 |  |  |  |  |
| 63 | Linear SVM | 0.8278146 | 0.847527 |  |  |  |  |
| 64 | Linear SVM | 0.8344371 | 0.874542 |  |  |  |  |
| 65 | Linear SVM | 0.8013245 | 0.826694 |  |  |  |  |
| 66 | Linear SVM | 0.8543046 | 0.871795 |  |  |  |  |
| 67 | Linear SVM | 0.8145695 | 0.865614 |  |  |  |  |
| 68 | Linear SVM | 0.8410596 | 0.89652 |  |  |  |  |
| 69 | Linear SVM | 0.8543046 | 0.914835 |  |  |  |  |
| 70 | Linear SVM | 0.8145695 | 0.845925 |  |  |  |  |
| 71 | Linear SVM | 0.8211921 | 0.861722 |  |  |  |  |
| 72 | Linear SVM | 0.8013245 | 0.824405 |  |  |  |  |
| 73 | Linear SVM | 0.807947 | 0.85016 |  |  |  |  |
| 74 | Linear SVM | 0.8211921 | 0.85348 |  |  |  |  |
| 75 | Linear SVM | 0.8145695 | 0.822344 |  |  |  |  |
| 76 | Linear SVM | 0.8013245 | 0.832189 |  |  |  |  |
| 77 | Linear SVM | 0.8278146 | 0.872024 |  |  |  |  |
| 78 | Linear SVM | 0.8410596 | 0.852793 |  |  |  |  |
| 79 | Linear SVM | 0.8013245 | 0.793956 |  |  |  |  |
| 80 | Linear SVM | 0.8675497 | 0.879121 |  |  |  |  |
| 81 | Linear SVM | 0.8344371 | 0.893544 |  |  |  |  |
| 82 | Linear SVM | 0.8145695 | 0.842033 |  |  |  |  |
| 83 | Linear SVM | 0.7880795 | 0.763049 |  |  |  |  |
| 84 | Linear SVM | 0.8278146 | 0.854625 |  |  |  |  |
| 85 | Linear SVM | 0.8278146 | 0.857143 |  |  |  |  |
| 86 | Linear SVM | 0.807947 | 0.842033 |  |  |  |  |
| 87 | Linear SVM | 0.8476821 | 0.826923 |  |  |  |  |
| 88 | Linear SVM | 0.8344371 | 0.87294 |  |  |  |  |
| 89 | Linear SVM | 0.8543046 | 0.85783 |  |  |  |  |
| 90 | Linear SVM | 0.807947 | 0.82326 |  |  |  |  |
| 91 | Linear SVM | 0.8211921 | 0.847299 |  |  |  |  |
| 92 | Linear SVM | 0.781457 | 0.866987 |  |  |  |  |
| 93 | Linear SVM | 0.794702 | 0.875 |  |  |  |  |
| 94 | Linear SVM | 0.807947 | 0.877289 |  |  |  |  |
| 95 | Linear SVM | 0.8278146 | 0.872482 |  |  |  |  |
| 96 | Linear SVM | 0.8344371 | 0.869048 |  |  |  |  |
| 97 | Linear SVM | 0.8344371 | 0.864927 |  |  |  |  |
| 98 | Linear SVM | 0.8211921 | 0.881296 |  |  |  |  |
| 99 | Linear SVM | 0.8145695 | 0.863324 |  |  |  |  |
| 100 | RBF SVM | 0.807947 | 0.79739 | 0.970 | 0.403 | 108.600 | 15.720 |
| 101 | RBF SVM | 0.8278146 | 0.834478 |  |  |  |  |
| 102 | RBF SVM | 0.8013245 | 0.80174 |  |  |  |  |
| 103 | RBF SVM | 0.781457 | 0.825778 |  |  |  |  |
| 104 | RBF SVM | 0.8609272 | 0.825549 |  |  |  |  |
| 105 | RBF SVM | 0.8013245 | 0.827839 |  |  |  |  |
| 106 | RBF SVM | 0.8278146 | 0.864698 |  |  |  |  |
| 107 | RBF SVM | 0.8344371 | 0.869734 |  |  |  |  |
| 108 | RBF SVM | 0.8013245 | 0.833104 |  |  |  |  |
| 109 | RBF SVM | 0.8344371 | 0.883929 |  |  |  |  |
| 110 | RBF SVM | 0.8013245 | 0.860348 |  |  |  |  |
| 111 | RBF SVM | 0.8476821 | 0.82967 |  |  |  |  |
| 112 | RBF SVM | 0.807947 | 0.770604 |  |  |  |  |
| 113 | RBF SVM | 0.807947 | 0.848901 |  |  |  |  |
| 114 | RBF SVM | 0.8013245 | 0.873626 |  |  |  |  |
| 115 | RBF SVM | 0.7880795 | 0.876145 |  |  |  |  |
| 116 | RBF SVM | 0.8476821 | 0.869391 |  |  |  |  |
| 117 | RBF SVM | 0.8410596 | 0.883471 |  |  |  |  |
| 118 | RBF SVM | 0.794702 | 0.825778 |  |  |  |  |
| 119 | RBF SVM | 0.8278146 | 0.851419 |  |  |  |  |
| 120 | RBF SVM | 0.8145695 | 0.90087 |  |  |  |  |
| 121 | RBF SVM | 0.8410596 | 0.857601 |  |  |  |  |
| 122 | RBF SVM | 0.8211921 | 0.8125 |  |  |  |  |
| 123 | RBF SVM | 0.8344371 | 0.846383 |  |  |  |  |
| 124 | RBF SVM | 0.8145695 | 0.861951 |  |  |  |  |
| 125 | RBF SVM | 0.8278146 | 0.86859 |  |  |  |  |
| 126 | RBF SVM | 0.807947 | 0.772436 |  |  |  |  |
| 127 | RBF SVM | 0.807947 | 0.846154 |  |  |  |  |
| 128 | RBF SVM | 0.807947 | 0.843864 |  |  |  |  |
| 129 | RBF SVM | 0.8013245 | 0.823489 |  |  |  |  |
| 130 | RBF SVM | 0.8211921 | 0.840659 |  |  |  |  |
| 131 | RBF SVM | 0.8278146 | 0.874886 |  |  |  |  |
| 132 | RBF SVM | 0.807947 | 0.809982 |  |  |  |  |
| 133 | RBF SVM | 0.8145695 | 0.923535 |  |  |  |  |
| 134 | RBF SVM | 0.8278146 | 0.878892 |  |  |  |  |
| 135 | RBF SVM | 0.8278146 | 0.793727 |  |  |  |  |
| 136 | RBF SVM | 0.8344371 | 0.862637 |  |  |  |  |
| 137 | RBF SVM | 0.781457 | 0.850962 |  |  |  |  |
| 138 | RBF SVM | 0.8278146 | 0.844437 |  |  |  |  |
| 139 | RBF SVM | 0.807947 | 0.835623 |  |  |  |  |
| 140 | RBF SVM | 0.8013245 | 0.829212 |  |  |  |  |
| 141 | RBF SVM | 0.7615894 | 0.797161 |  |  |  |  |
| 142 | RBF SVM | 0.8013245 | 0.796932 |  |  |  |  |
| 143 | RBF SVM | 0.8211921 | 0.841804 |  |  |  |  |
| 144 | RBF SVM | 0.8410596 | 0.860348 |  |  |  |  |
| 145 | RBF SVM | 0.794702 | 0.82967 |  |  |  |  |
| 146 | RBF SVM | 0.7880795 | 0.831044 |  |  |  |  |
| 147 | RBF SVM | 0.8278146 | 0.829441 |  |  |  |  |
| 148 | RBF SVM | 0.8476821 | 0.863782 |  |  |  |  |
| 149 | RBF SVM | 0.8543046 | 0.910027 |  |  |  |  |
| 150 | Gaussian Process | 0.7417219 | 0.84043 | 1.000 | 0.000 | 112.000 | 0.000 |
| 151 | Gaussian Process | 0.7417219 | 0.87935 |  |  |  |  |
| 152 | Gaussian Process | 0.7417219 | 0.826465 |  |  |  |  |
| 153 | Gaussian Process | 0.7417219 | 0.816163 |  |  |  |  |
| 154 | Gaussian Process | 0.7417219 | 0.803114 |  |  |  |  |
| 155 | Gaussian Process | 0.7417219 | 0.815934 |  |  |  |  |
| 156 | Gaussian Process | 0.7417219 | 0.830815 |  |  |  |  |
| 157 | Gaussian Process | 0.7417219 | 0.743132 |  |  |  |  |
| 158 | Gaussian Process | 0.7417219 | 0.803571 |  |  |  |  |
| 159 | Gaussian Process | 0.7417219 | 0.821886 |  |  |  |  |
| 160 | Gaussian Process | 0.7417219 | 0.828068 |  |  |  |  |
| 161 | Gaussian Process | 0.7417219 | 0.866529 |  |  |  |  |
| 162 | Gaussian Process | 0.7417219 | 0.841346 |  |  |  |  |
| 163 | Gaussian Process | 0.7417219 | 0.827381 |  |  |  |  |
| 164 | Gaussian Process | 0.7417219 | 0.815934 |  |  |  |  |
| 165 | Gaussian Process | 0.7417219 | 0.781364 |  |  |  |  |
| 166 | Gaussian Process | 0.7417219 | 0.863553 |  |  |  |  |
| 167 | Gaussian Process | 0.7417219 | 0.835623 |  |  |  |  |
| 168 | Gaussian Process | 0.7417219 | 0.832418 |  |  |  |  |
| 169 | Gaussian Process | 0.7417219 | 0.811355 |  |  |  |  |
| 170 | Gaussian Process | 0.7417219 | 0.851877 |  |  |  |  |
| 171 | Gaussian Process | 0.7417219 | 0.831044 |  |  |  |  |
| 172 | Gaussian Process | 0.7417219 | 0.82967 |  |  |  |  |
| 173 | Gaussian Process | 0.7417219 | 0.800595 |  |  |  |  |
| 174 | Gaussian Process | 0.7417219 | 0.861035 |  |  |  |  |
| 175 | Gaussian Process | 0.7417219 | 0.871566 |  |  |  |  |
| 176 | Gaussian Process | 0.7417219 | 0.836081 |  |  |  |  |
| 177 | Gaussian Process | 0.7417219 | 0.893544 |  |  |  |  |
| 178 | Gaussian Process | 0.7417219 | 0.820742 |  |  |  |  |
| 179 | Gaussian Process | 0.7417219 | 0.79533 |  |  |  |  |
| 180 | Gaussian Process | 0.7417219 | 0.872482 |  |  |  |  |
| 181 | Gaussian Process | 0.7417219 | 0.854853 |  |  |  |  |
| 182 | Gaussian Process | 0.7417219 | 0.820971 |  |  |  |  |
| 183 | Gaussian Process | 0.7417219 | 0.811584 |  |  |  |  |
| 184 | Gaussian Process | 0.7417219 | 0.83837 |  |  |  |  |
| 185 | Gaussian Process | 0.7417219 | 0.802885 |  |  |  |  |
| 186 | Gaussian Process | 0.7417219 | 0.875229 |  |  |  |  |
| 187 | Gaussian Process | 0.7417219 | 0.790751 |  |  |  |  |
| 188 | Gaussian Process | 0.7417219 | 0.809753 |  |  |  |  |
| 189 | Gaussian Process | 0.7417219 | 0.86424 |  |  |  |  |
| 190 | Gaussian Process | 0.7417219 | 0.888507 |  |  |  |  |
| 191 | Gaussian Process | 0.7417219 | 0.80815 |  |  |  |  |
| 192 | Gaussian Process | 0.7417219 | 0.807692 |  |  |  |  |
| 193 | Gaussian Process | 0.7417219 | 0.834478 |  |  |  |  |
| 194 | Gaussian Process | 0.7417219 | 0.804945 |  |  |  |  |
| 195 | Gaussian Process | 0.7417219 | 0.856227 |  |  |  |  |
| 196 | Gaussian Process | 0.7417219 | 0.850275 |  |  |  |  |
| 197 | Gaussian Process | 0.7417219 | 0.763736 |  |  |  |  |
| 198 | Gaussian Process | 0.7417219 | 0.826236 |  |  |  |  |
| 199 | Gaussian Process | 0.7417219 | 0.839057 |  |  |  |  |
| 200 | Random Forest | 0.8145695 | 0.85554 | 0.952 | 0.436 | 106.640 | 17.000 |
| 201 | Random Forest | 0.7880795 | 0.80815 |  |  |  |  |
| 202 | Random Forest | 0.8344371 | 0.873168 |  |  |  |  |
| 203 | Random Forest | 0.807947 | 0.794643 |  |  |  |  |
| 204 | Random Forest | 0.8278146 | 0.880952 |  |  |  |  |
| 205 | Random Forest | 0.7880795 | 0.791667 |  |  |  |  |
| 206 | Random Forest | 0.807947 | 0.838141 |  |  |  |  |
| 207 | Random Forest | 0.807947 | 0.863553 |  |  |  |  |
| 208 | Random Forest | 0.8410596 | 0.85554 |  |  |  |  |
| 209 | Random Forest | 0.8145695 | 0.818681 |  |  |  |  |
| 210 | Random Forest | 0.794702 | 0.845009 |  |  |  |  |
| 211 | Random Forest | 0.807947 | 0.799451 |  |  |  |  |
| 212 | Random Forest | 0.8609272 | 0.894689 |  |  |  |  |
| 213 | Random Forest | 0.8543046 | 0.847756 |  |  |  |  |
| 214 | Random Forest | 0.8145695 | 0.835623 |  |  |  |  |
| 215 | Random Forest | 0.8807947 | 0.872253 |  |  |  |  |
| 216 | Random Forest | 0.794702 | 0.803571 |  |  |  |  |
| 217 | Random Forest | 0.8344371 | 0.85348 |  |  |  |  |
| 218 | Random Forest | 0.8013245 | 0.821886 |  |  |  |  |
| 219 | Random Forest | 0.794702 | 0.807005 |  |  |  |  |
| 220 | Random Forest | 0.7880795 | 0.815705 |  |  |  |  |
| 221 | Random Forest | 0.7880795 | 0.796245 |  |  |  |  |
| 222 | Random Forest | 0.8410596 | 0.824863 |  |  |  |  |
| 223 | Random Forest | 0.8211921 | 0.845238 |  |  |  |  |
| 224 | Random Forest | 0.8675497 | 0.882326 |  |  |  |  |
| 225 | Random Forest | 0.8476821 | 0.849817 |  |  |  |  |
| 226 | Random Forest | 0.7880795 | 0.789148 |  |  |  |  |
| 227 | Random Forest | 0.8410596 | 0.863553 |  |  |  |  |
| 228 | Random Forest | 0.8211921 | 0.879579 |  |  |  |  |
| 229 | Random Forest | 0.8410596 | 0.849588 |  |  |  |  |
| 230 | Random Forest | 0.8476821 | 0.877747 |  |  |  |  |
| 231 | Random Forest | 0.8476821 | 0.868361 |  |  |  |  |
| 232 | Random Forest | 0.8211921 | 0.80174 |  |  |  |  |
| 233 | Random Forest | 0.8145695 | 0.81685 |  |  |  |  |
| 234 | Random Forest | 0.8278146 | 0.856914 |  |  |  |  |
| 235 | Random Forest | 0.8013245 | 0.829212 |  |  |  |  |
| 236 | Random Forest | 0.8278146 | 0.842491 |  |  |  |  |
| 237 | Random Forest | 0.8145695 | 0.883471 |  |  |  |  |
| 238 | Random Forest | 0.807947 | 0.856227 |  |  |  |  |
| 239 | Random Forest | 0.8013245 | 0.843407 |  |  |  |  |
| 240 | Random Forest | 0.807947 | 0.878892 |  |  |  |  |
| 241 | Random Forest | 0.8476821 | 0.87065 |  |  |  |  |
| 242 | Random Forest | 0.794702 | 0.815247 |  |  |  |  |
| 243 | Random Forest | 0.8145695 | 0.786172 |  |  |  |  |
| 244 | Random Forest | 0.8278146 | 0.850275 |  |  |  |  |
| 245 | Random Forest | 0.8344371 | 0.85554 |  |  |  |  |
| 246 | Random Forest | 0.8278146 | 0.863324 |  |  |  |  |
| 247 | Random Forest | 0.8476821 | 0.855998 |  |  |  |  |
| 248 | Random Forest | 0.8278146 | 0.850046 |  |  |  |  |
| 249 | Random Forest | 0.8344371 | 0.823718 |  |  |  |  |
| 250 | Neural Net | 0.781457 | 0.810668 | 0.898 | 0.533 | 100.540 | 20.780 |
| 251 | Neural Net | 0.8410596 | 0.83837 |  |  |  |  |
| 252 | Neural Net | 0.794702 | 0.837225 |  |  |  |  |
| 253 | Neural Net | 0.781457 | 0.809982 |  |  |  |  |
| 254 | Neural Net | 0.7748344 | 0.80815 |  |  |  |  |
| 255 | Neural Net | 0.807947 | 0.824863 |  |  |  |  |
| 256 | Neural Net | 0.794702 | 0.801282 |  |  |  |  |
| 257 | Neural Net | 0.7880795 | 0.763507 |  |  |  |  |
| 258 | Neural Net | 0.781457 | 0.787775 |  |  |  |  |
| 259 | Neural Net | 0.8013245 | 0.836081 |  |  |  |  |
| 260 | Neural Net | 0.8278146 | 0.833333 |  |  |  |  |
| 261 | Neural Net | 0.8278146 | 0.85119 |  |  |  |  |
| 262 | Neural Net | 0.8013245 | 0.801282 |  |  |  |  |
| 263 | Neural Net | 0.7417219 | 0.79533 |  |  |  |  |
| 264 | Neural Net | 0.8013245 | 0.833104 |  |  |  |  |
| 265 | Neural Net | 0.794702 | 0.828526 |  |  |  |  |
| 266 | Neural Net | 0.8278146 | 0.845467 |  |  |  |  |
| 267 | Neural Net | 0.8211921 | 0.825092 |  |  |  |  |
| 268 | Neural Net | 0.8013245 | 0.832647 |  |  |  |  |
| 269 | Neural Net | 0.8278146 | 0.817079 |  |  |  |  |
| 270 | Neural Net | 0.794702 | 0.839973 |  |  |  |  |
| 271 | Neural Net | 0.8013245 | 0.733059 |  |  |  |  |
| 272 | Neural Net | 0.7748344 | 0.761218 |  |  |  |  |
| 273 | Neural Net | 0.8211921 | 0.841117 |  |  |  |  |
| 274 | Neural Net | 0.7483444 | 0.767628 |  |  |  |  |
| 275 | Neural Net | 0.794702 | 0.831731 |  |  |  |  |
| 276 | Neural Net | 0.8211921 | 0.807692 |  |  |  |  |
| 277 | Neural Net | 0.794702 | 0.837683 |  |  |  |  |
| 278 | Neural Net | 0.8013245 | 0.828068 |  |  |  |  |
| 279 | Neural Net | 0.7483444 | 0.800824 |  |  |  |  |
| 280 | Neural Net | 0.8211921 | 0.835852 |  |  |  |  |
| 281 | Neural Net | 0.7350993 | 0.789606 |  |  |  |  |
| 282 | Neural Net | 0.7615894 | 0.738324 |  |  |  |  |
| 283 | Neural Net | 0.8145695 | 0.85348 |  |  |  |  |
| 284 | Neural Net | 0.794702 | 0.804029 |  |  |  |  |
| 285 | Neural Net | 0.781457 | 0.841575 |  |  |  |  |
| 286 | Neural Net | 0.7880795 | 0.759844 |  |  |  |  |
| 287 | Neural Net | 0.7483444 | 0.761218 |  |  |  |  |
| 288 | Neural Net | 0.7880795 | 0.764881 |  |  |  |  |
| 289 | Neural Net | 0.7880795 | 0.809524 |  |  |  |  |
| 290 | Neural Net | 0.8278146 | 0.862866 |  |  |  |  |
| 291 | Neural Net | 0.807947 | 0.848214 |  |  |  |  |
| 292 | Neural Net | 0.8476821 | 0.885531 |  |  |  |  |
| 293 | Neural Net | 0.8013245 | 0.792811 |  |  |  |  |
| 294 | Neural Net | 0.8476821 | 0.812042 |  |  |  |  |
| 295 | Neural Net | 0.8013245 | 0.794414 |  |  |  |  |
| 296 | Neural Net | 0.8476821 | 0.884844 |  |  |  |  |
| 297 | Neural Net | 0.8476821 | 0.848901 |  |  |  |  |
| 298 | Neural Net | 0.8344371 | 0.87706 |  |  |  |  |
| 299 | Neural Net | 0.8278146 | 0.877518 |  |  |  |  |
| 300 | AdaBoost | 0.7549669 | 0.729167 | 0.887 | 0.536 | 99.320 | 20.920 |
| 301 | AdaBoost | 0.781457 | 0.772665 |  |  |  |  |
| 302 | AdaBoost | 0.807947 | 0.806891 |  |  |  |  |
| 303 | AdaBoost | 0.7748344 | 0.768773 |  |  |  |  |
| 304 | AdaBoost | 0.781457 | 0.777587 |  |  |  |  |
| 305 | AdaBoost | 0.7880795 | 0.824061 |  |  |  |  |
| 306 | AdaBoost | 0.7549669 | 0.764309 |  |  |  |  |
| 307 | AdaBoost | 0.794702 | 0.743704 |  |  |  |  |
| 308 | AdaBoost | 0.8145695 | 0.830357 |  |  |  |  |
| 309 | AdaBoost | 0.794702 | 0.800023 |  |  |  |  |
| 310 | AdaBoost | 0.7880795 | 0.788233 |  |  |  |  |
| 311 | AdaBoost | 0.8278146 | 0.771291 |  |  |  |  |
| 312 | AdaBoost | 0.8211921 | 0.755952 |  |  |  |  |
| 313 | AdaBoost | 0.8410596 | 0.821085 |  |  |  |  |
| 314 | AdaBoost | 0.8543046 | 0.877976 |  |  |  |  |
| 315 | AdaBoost | 0.7880795 | 0.765339 |  |  |  |  |
| 316 | AdaBoost | 0.7086093 | 0.711195 |  |  |  |  |
| 317 | AdaBoost | 0.794702 | 0.763393 |  |  |  |  |
| 318 | AdaBoost | 0.8013245 | 0.820971 |  |  |  |  |
| 319 | AdaBoost | 0.7483444 | 0.74794 |  |  |  |  |
| 320 | AdaBoost | 0.8013245 | 0.791438 |  |  |  |  |
| 321 | AdaBoost | 0.8543046 | 0.869505 |  |  |  |  |
| 322 | AdaBoost | 0.7682119 | 0.744849 |  |  |  |  |
| 323 | AdaBoost | 0.7682119 | 0.757555 |  |  |  |  |
| 324 | AdaBoost | 0.8013245 | 0.828297 |  |  |  |  |
| 325 | AdaBoost | 0.781457 | 0.825778 |  |  |  |  |
| 326 | AdaBoost | 0.8145695 | 0.828984 |  |  |  |  |
| 327 | AdaBoost | 0.807947 | 0.838141 |  |  |  |  |
| 328 | AdaBoost | 0.8278146 | 0.807005 |  |  |  |  |
| 329 | AdaBoost | 0.781457 | 0.801854 |  |  |  |  |
| 330 | AdaBoost | 0.7748344 | 0.78022 |  |  |  |  |
| 331 | AdaBoost | 0.8741722 | 0.840888 |  |  |  |  |
| 332 | AdaBoost | 0.8278146 | 0.801053 |  |  |  |  |
| 333 | AdaBoost | 0.7417219 | 0.769231 |  |  |  |  |
| 334 | AdaBoost | 0.807947 | 0.853938 |  |  |  |  |
| 335 | AdaBoost | 0.807947 | 0.804373 |  |  |  |  |
| 336 | AdaBoost | 0.8741722 | 0.868132 |  |  |  |  |
| 337 | AdaBoost | 0.8675497 | 0.846726 |  |  |  |  |
| 338 | AdaBoost | 0.8476821 | 0.82967 |  |  |  |  |
| 339 | AdaBoost | 0.7218543 | 0.732028 |  |  |  |  |
| 340 | AdaBoost | 0.8211921 | 0.78331 |  |  |  |  |
| 341 | AdaBoost | 0.7549669 | 0.792239 |  |  |  |  |
| 342 | AdaBoost | 0.7880795 | 0.76717 |  |  |  |  |
| 343 | AdaBoost | 0.8211921 | 0.83299 |  |  |  |  |
| 344 | AdaBoost | 0.781457 | 0.749084 |  |  |  |  |
| 345 | AdaBoost | 0.781457 | 0.769231 |  |  |  |  |
| 346 | AdaBoost | 0.8278146 | 0.846612 |  |  |  |  |
| 347 | AdaBoost | 0.794702 | 0.805632 |  |  |  |  |
| 348 | AdaBoost | 0.781457 | 0.761561 |  |  |  |  |
| 349 | AdaBoost | 0.7549669 | 0.772321 |  |  |  |  |
| 350 | Naive Bayes | 0.7086093 | 0.778732 | 0.737 | 0.792 | 82.500 | 30.880 |
| 351 | Naive Bayes | 0.6225166 | 0.794185 |  |  |  |  |
| 352 | Naive Bayes | 0.6291391 | 0.839744 |  |  |  |  |
| 353 | Naive Bayes | 0.6225166 | 0.815247 |  |  |  |  |
| 354 | Naive Bayes | 0.7880795 | 0.875687 |  |  |  |  |
| 355 | Naive Bayes | 0.7615894 | 0.855311 |  |  |  |  |
| 356 | Naive Bayes | 0.7086093 | 0.835165 |  |  |  |  |
| 357 | Naive Bayes | 0.8013245 | 0.882097 |  |  |  |  |
| 358 | Naive Bayes | 0.7417219 | 0.832647 |  |  |  |  |
| 359 | Naive Bayes | 0.6953642 | 0.782395 |  |  |  |  |
| 360 | Naive Bayes | 0.781457 | 0.875343 |  |  |  |  |
| 361 | Naive Bayes | 0.7152318 | 0.892285 |  |  |  |  |
| 362 | Naive Bayes | 0.7284768 | 0.839057 |  |  |  |  |
| 363 | Naive Bayes | 0.6490066 | 0.814789 |  |  |  |  |
| 364 | Naive Bayes | 0.794702 | 0.852106 |  |  |  |  |
| 365 | Naive Bayes | 0.7350993 | 0.850046 |  |  |  |  |
| 366 | Naive Bayes | 0.7284768 | 0.840774 |  |  |  |  |
| 367 | Naive Bayes | 0.7682119 | 0.849245 |  |  |  |  |
| 368 | Naive Bayes | 0.7682119 | 0.813759 |  |  |  |  |
| 369 | Naive Bayes | 0.7748344 | 0.844093 |  |  |  |  |
| 370 | Naive Bayes | 0.7417219 | 0.820284 |  |  |  |  |
| 371 | Naive Bayes | 0.7350993 | 0.807005 |  |  |  |  |
| 372 | Naive Bayes | 0.7549669 | 0.805288 |  |  |  |  |
| 373 | Naive Bayes | 0.7152318 | 0.864354 |  |  |  |  |
| 374 | Naive Bayes | 0.7417219 | 0.846612 |  |  |  |  |
| 375 | Naive Bayes | 0.7615894 | 0.845925 |  |  |  |  |
| 376 | Naive Bayes | 0.7483444 | 0.860348 |  |  |  |  |
| 377 | Naive Bayes | 0.7748344 | 0.836081 |  |  |  |  |
| 378 | Naive Bayes | 0.7218543 | 0.828411 |  |  |  |  |
| 379 | Naive Bayes | 0.8344371 | 0.928114 |  |  |  |  |
| 380 | Naive Bayes | 0.794702 | 0.904533 |  |  |  |  |
| 381 | Naive Bayes | 0.7417219 | 0.867903 |  |  |  |  |
| 382 | Naive Bayes | 0.7682119 | 0.822802 |  |  |  |  |
| 383 | Naive Bayes | 0.8013245 | 0.860577 |  |  |  |  |
| 384 | Naive Bayes | 0.794702 | 0.862866 |  |  |  |  |
| 385 | Naive Bayes | 0.7417219 | 0.831044 |  |  |  |  |
| 386 | Naive Bayes | 0.7417219 | 0.838942 |  |  |  |  |
| 387 | Naive Bayes | 0.6490066 | 0.825778 |  |  |  |  |
| 388 | Naive Bayes | 0.7682119 | 0.858059 |  |  |  |  |
| 389 | Naive Bayes | 0.7019868 | 0.832418 |  |  |  |  |
| 390 | Naive Bayes | 0.6754967 | 0.832189 |  |  |  |  |
| 391 | Naive Bayes | 0.794702 | 0.826694 |  |  |  |  |
| 392 | Naive Bayes | 0.6754967 | 0.857601 |  |  |  |  |
| 393 | Naive Bayes | 0.6490066 | 0.835852 |  |  |  |  |
| 394 | Naive Bayes | 0.7284768 | 0.834936 |  |  |  |  |
| 395 | Naive Bayes | 0.781457 | 0.887134 |  |  |  |  |
| 396 | Naive Bayes | 0.7483444 | 0.810554 |  |  |  |  |
| 397 | Naive Bayes | 0.7682119 | 0.840316 |  |  |  |  |
| 398 | Naive Bayes | 0.8013245 | 0.882555 |  |  |  |  |
| 399 | Naive Bayes | 0.794702 | 0.860119 |  |  |  |  |

| *Table 2. Fifty-times iteration of models for Steatosis Stage.* | | | |
| --- | --- | --- | --- |
| *Column1* | ***ClfName*** | ***Accuracy*** | ***AUC*** |
| *0* | *Nearest Neighbors* | *0.4172185* | *0.627452* |
| *1* | *Nearest Neighbors* | *0.3642384* | *0.606214* |
| *2* | *Nearest Neighbors* | *0.3907285* | *0.596642* |
| *3* | *Nearest Neighbors* | *0.4304636* | *0.6007* |
| *4* | *Nearest Neighbors* | *0.3907285* | *0.62215* |
| *5* | *Nearest Neighbors* | *0.410596* | *0.615482* |
| *6* | *Nearest Neighbors* | *0.4768212* | *0.649397* |
| *7* | *Nearest Neighbors* | *0.3774834* | *0.613508* |
| *8* | *Nearest Neighbors* | *0.3576159* | *0.628507* |
| *9* | *Nearest Neighbors* | *0.410596* | *0.609357* |
| *10* | *Nearest Neighbors* | *0.4238411* | *0.593054* |
| *11* | *Nearest Neighbors* | *0.3443709* | *0.585468* |
| *12* | *Nearest Neighbors* | *0.410596* | *0.625042* |
| *13* | *Nearest Neighbors* | *0.3642384* | *0.566162* |
| *14* | *Nearest Neighbors* | *0.4304636* | *0.627319* |
| *15* | *Nearest Neighbors* | *0.4437086* | *0.660016* |
| *16* | *Nearest Neighbors* | *0.3642384* | *0.587246* |
| *17* | *Nearest Neighbors* | *0.3708609* | *0.626037* |
| *18* | *Nearest Neighbors* | *0.4039735* | *0.624852* |
| *19* | *Nearest Neighbors* | *0.384106* | *0.567774* |
| *20* | *Nearest Neighbors* | *0.3708609* | *0.631887* |
| *21* | *Nearest Neighbors* | *0.3576159* | *0.60516* |
| *22* | *Nearest Neighbors* | *0.3642384* | *0.614377* |
| *23* | *Nearest Neighbors* | *0.4701987* | *0.645427* |
| *24* | *Nearest Neighbors* | *0.410596* | *0.619004* |
| *25* | *Nearest Neighbors* | *0.3774834* | *0.593814* |
| *26* | *Nearest Neighbors* | *0.4172185* | *0.632061* |
| *27* | *Nearest Neighbors* | *0.384106* | *0.586201* |
| *28* | *Nearest Neighbors* | *0.4304636* | *0.65878* |
| *29* | *Nearest Neighbors* | *0.397351* | *0.62852* |
| *30* | *Nearest Neighbors* | *0.3509934* | *0.624219* |
| *31* | *Nearest Neighbors* | *0.410596* | *0.614521* |
| *32* | *Nearest Neighbors* | *0.4370861* | *0.682958* |
| *33* | *Nearest Neighbors* | *0.4039735* | *0.600456* |
| *34* | *Nearest Neighbors* | *0.4370861* | *0.645196* |
| *35* | *Nearest Neighbors* | *0.3377483* | *0.575812* |
| *36* | *Nearest Neighbors* | *0.3774834* | *0.601023* |
| *37* | *Nearest Neighbors* | *0.4900662* | *0.674859* |
| *38* | *Nearest Neighbors* | *0.4238411* | *0.621914* |
| *39* | *Nearest Neighbors* | *0.4635762* | *0.651992* |
| *40* | *Nearest Neighbors* | *0.384106* | *0.592492* |
| *41* | *Nearest Neighbors* | *0.3708609* | *0.615787* |
| *42* | *Nearest Neighbors* | *0.397351* | *0.626897* |
| *43* | *Nearest Neighbors* | *0.397351* | *0.635601* |
| *44* | *Nearest Neighbors* | *0.3509934* | *0.573405* |
| *45* | *Nearest Neighbors* | *0.410596* | *0.652773* |
| *46* | *Nearest Neighbors* | *0.4238411* | *0.610714* |
| *47* | *Nearest Neighbors* | *0.3377483* | *0.58657* |
| *48* | *Nearest Neighbors* | *0.410596* | *0.619517* |
| *49* | *Nearest Neighbors* | *0.4172185* | *0.635885* |
| *50* | *Linear SVM* | *0.5231788* | *0.677528* |
| *51* | *Linear SVM* | *0.5364238* | *0.678447* |
| *52* | *Linear SVM* | *0.5629139* | *0.720702* |
| *53* | *Linear SVM* | *0.5364238* | *0.669637* |
| *54* | *Linear SVM* | *0.5298013* | *0.689584* |
| *55* | *Linear SVM* | *0.5364238* | *0.71369* |
| *56* | *Linear SVM* | *0.5231788* | *0.674222* |
| *57* | *Linear SVM* | *0.5033113* | *0.696748* |
| *58* | *Linear SVM* | *0.5364238* | *0.658039* |
| *59* | *Linear SVM* | *0.5364238* | *0.703915* |
| *60* | *Linear SVM* | *0.5298013* | *0.6882* |
| *61* | *Linear SVM* | *0.5231788* | *0.683767* |
| *62* | *Linear SVM* | *0.5629139* | *0.683138* |
| *63* | *Linear SVM* | *0.5298013* | *0.69991* |
| *64* | *Linear SVM* | *0.5629139* | *0.750023* |
| *65* | *Linear SVM* | *0.5364238* | *0.720978* |
| *66* | *Linear SVM* | *0.5033113* | *0.658525* |
| *67* | *Linear SVM* | *0.5364238* | *0.686966* |
| *68* | *Linear SVM* | *0.5430464* | *0.682283* |
| *69* | *Linear SVM* | *0.5364238* | *0.705265* |
| *70* | *Linear SVM* | *0.4966887* | *0.675985* |
| *71* | *Linear SVM* | *0.4834437* | *0.65876* |
| *72* | *Linear SVM* | *0.5231788* | *0.650755* |
| *73* | *Linear SVM* | *0.5165563* | *0.665543* |
| *74* | *Linear SVM* | *0.5496689* | *0.717745* |
| *75* | *Linear SVM* | *0.5231788* | *0.732322* |
| *76* | *Linear SVM* | *0.5033113* | *0.676356* |
| *77* | *Linear SVM* | *0.5695364* | *0.703411* |
| *78* | *Linear SVM* | *0.5364238* | *0.679668* |
| *79* | *Linear SVM* | *0.5231788* | *0.67616* |
| *80* | *Linear SVM* | *0.5496689* | *0.690398* |
| *81* | *Linear SVM* | *0.5298013* | *0.713079* |
| *82* | *Linear SVM* | *0.5562914* | *0.698139* |
| *83* | *Linear SVM* | *0.5231788* | *0.72197* |
| *84* | *Linear SVM* | *0.5231788* | *0.687534* |
| *85* | *Linear SVM* | *0.5165563* | *0.676757* |
| *86* | *Linear SVM* | *0.5629139* | *0.69566* |
| *87* | *Linear SVM* | *0.5165563* | *0.715562* |
| *88* | *Linear SVM* | *0.5364238* | *0.679914* |
| *89* | *Linear SVM* | *0.5629139* | *0.727407* |
| *90* | *Linear SVM* | *0.5562914* | *0.724963* |
| *91* | *Linear SVM* | *0.5033113* | *0.699085* |
| *92* | *Linear SVM* | *0.5364238* | *0.698049* |
| *93* | *Linear SVM* | *0.5761589* | *0.712718* |
| *94* | *Linear SVM* | *0.5562914* | *0.661654* |
| *95* | *Linear SVM* | *0.5562914* | *0.722064* |
| *96* | *Linear SVM* | *0.5496689* | *0.672909* |
| *97* | *Linear SVM* | *0.5430464* | *0.667847* |
| *98* | *Linear SVM* | *0.4900662* | *0.67115* |
| *99* | *Linear SVM* | *0.5231788* | *0.676182* |
| *100* | *RBF SVM* | *0.5298013* | *0.67638* |
| *101* | *RBF SVM* | *0.5496689* | *0.708454* |
| *102* | *RBF SVM* | *0.4834437* | *0.635176* |
| *103* | *RBF SVM* | *0.5364238* | *0.672911* |
| *104* | *RBF SVM* | *0.4701987* | *0.661378* |
| *105* | *RBF SVM* | *0.5364238* | *0.668888* |
| *106* | *RBF SVM* | *0.5562914* | *0.679145* |
| *107* | *RBF SVM* | *0.5099338* | *0.658276* |
| *108* | *RBF SVM* | *0.5033113* | *0.682881* |
| *109* | *RBF SVM* | *0.5099338* | *0.630314* |
| *110* | *RBF SVM* | *0.5364238* | *0.68701* |
| *111* | *RBF SVM* | *0.4966887* | *0.669731* |
| *112* | *RBF SVM* | *0.5033113* | *0.620923* |
| *113* | *RBF SVM* | *0.5695364* | *0.709176* |
| *114* | *RBF SVM* | *0.5629139* | *0.682893* |
| *115* | *RBF SVM* | *0.5298013* | *0.713583* |
| *116* | *RBF SVM* | *0.5761589* | *0.681951* |
| *117* | *RBF SVM* | *0.5364238* | *0.695373* |
| *118* | *RBF SVM* | *0.5298013* | *0.681056* |
| *119* | *RBF SVM* | *0.5165563* | *0.668929* |
| *120* | *RBF SVM* | *0.5364238* | *0.650446* |
| *121* | *RBF SVM* | *0.5165563* | *0.667989* |
| *122* | *RBF SVM* | *0.5562914* | *0.698825* |
| *123* | *RBF SVM* | *0.5695364* | *0.718005* |
| *124* | *RBF SVM* | *0.5099338* | *0.658068* |
| *125* | *RBF SVM* | *0.5298013* | *0.673834* |
| *126* | *RBF SVM* | *0.4768212* | *0.664308* |
| *127* | *RBF SVM* | *0.5298013* | *0.676301* |
| *128* | *RBF SVM* | *0.5099338* | *0.668016* |
| *129* | *RBF SVM* | *0.5165563* | *0.67694* |
| *130* | *RBF SVM* | *0.5496689* | *0.698095* |
| *131* | *RBF SVM* | *0.5364238* | *0.683018* |
| *132* | *RBF SVM* | *0.4966887* | *0.62814* |
| *133* | *RBF SVM* | *0.5562914* | *0.696678* |
| *134* | *RBF SVM* | *0.5562914* | *0.670859* |
| *135* | *RBF SVM* | *0.5496689* | *0.679396* |
| *136* | *RBF SVM* | *0.4966887* | *0.668105* |
| *137* | *RBF SVM* | *0.4834437* | *0.647469* |
| *138* | *RBF SVM* | *0.5033113* | *0.728529* |
| *139* | *RBF SVM* | *0.5364238* | *0.672441* |
| *140* | *RBF SVM* | *0.5231788* | *0.695387* |
| *141* | *RBF SVM* | *0.5033113* | *0.677878* |
| *142* | *RBF SVM* | *0.5298013* | *0.716042* |
| *143* | *RBF SVM* | *0.4966887* | *0.639375* |
| *144* | *RBF SVM* | *0.5430464* | *0.698199* |
| *145* | *RBF SVM* | *0.5231788* | *0.680012* |
| *146* | *RBF SVM* | *0.5430464* | *0.680493* |
| *147* | *RBF SVM* | *0.5298013* | *0.704329* |
| *148* | *RBF SVM* | *0.5364238* | *0.69962* |
| *149* | *RBF SVM* | *0.5496689* | *0.696591* |
| *150* | *Gaussian Process* | *0.5496689* | *0.636016* |
| *151* | *Gaussian Process* | *0.5165563* | *0.626193* |
| *152* | *Gaussian Process* | *0.5033113* | *0.627638* |
| *153* | *Gaussian Process* | *0.5827815* | *0.666223* |
| *154* | *Gaussian Process* | *0.397351* | *0.697291* |
| *155* | *Gaussian Process* | *0.5298013* | *0.625395* |
| *156* | *Gaussian Process* | *0.397351* | *0.67614* |
| *157* | *Gaussian Process* | *0.5496689* | *0.622583* |
| *158* | *Gaussian Process* | *0.5430464* | *0.651789* |
| *159* | *Gaussian Process* | *0.4834437* | *0.595408* |
| *160* | *Gaussian Process* | *0.5430464* | *0.628253* |
| *161* | *Gaussian Process* | *0.5496689* | *0.639033* |
| *162* | *Gaussian Process* | *0.397351* | *0.678677* |
| *163* | *Gaussian Process* | *0.5231788* | *0.647725* |
| *164* | *Gaussian Process* | *0.5231788* | *0.638607* |
| *165* | *Gaussian Process* | *0.5099338* | *0.645274* |
| *166* | *Gaussian Process* | *0.5629139* | *0.65361* |
| *167* | *Gaussian Process* | *0.5364238* | *0.634996* |
| *168* | *Gaussian Process* | *0.5496689* | *0.648052* |
| *169* | *Gaussian Process* | *0.5496689* | *0.643883* |
| *170* | *Gaussian Process* | *0.5165563* | *0.645764* |
| *171* | *Gaussian Process* | *0.5231788* | *0.629389* |
| *172* | *Gaussian Process* | *0.5629139* | *0.656315* |
| *173* | *Gaussian Process* | *0.5364238* | *0.63427* |
| *174* | *Gaussian Process* | *0.5364238* | *0.653732* |
| *175* | *Gaussian Process* | *0.5430464* | *0.642109* |
| *176* | *Gaussian Process* | *0.5165563* | *0.644421* |
| *177* | *Gaussian Process* | *0.5629139* | *0.660657* |
| *178* | *Gaussian Process* | *0.5695364* | *0.675166* |
| *179* | *Gaussian Process* | *0.5364238* | *0.619859* |
| *180* | *Gaussian Process* | *0.397351* | *0.677555* |
| *181* | *Gaussian Process* | *0.397351* | *0.669504* |
| *182* | *Gaussian Process* | *0.5430464* | *0.647175* |
| *183* | *Gaussian Process* | *0.5430464* | *0.631783* |
| *184* | *Gaussian Process* | *0.5496689* | *0.615906* |
| *185* | *Gaussian Process* | *0.5165563* | *0.632104* |
| *186* | *Gaussian Process* | *0.4966887* | *0.615897* |
| *187* | *Gaussian Process* | *0.5165563* | *0.618002* |
| *188* | *Gaussian Process* | *0.5165563* | *0.600905* |
| *189* | *Gaussian Process* | *0.397351* | *0.689367* |
| *190* | *Gaussian Process* | *0.5231788* | *0.630901* |
| *191* | *Gaussian Process* | *0.5629139* | *0.640797* |
| *192* | *Gaussian Process* | *0.397351* | *0.659038* |
| *193* | *Gaussian Process* | *0.5033113* | *0.632614* |
| *194* | *Gaussian Process* | *0.397351* | *0.679477* |
| *195* | *Gaussian Process* | *0.5298013* | *0.629241* |
| *196* | *Gaussian Process* | *0.397351* | *0.680211* |
| *197* | *Gaussian Process* | *0.5231788* | *0.646899* |
| *198* | *Gaussian Process* | *0.4966887* | *0.626552* |
| *199* | *Gaussian Process* | *0.5629139* | *0.61894* |
| *200* | *Random Forest* | *0.5364238* | *0.688484* |
| *201* | *Random Forest* | *0.5562914* | *0.710901* |
| *202* | *Random Forest* | *0.5231788* | *0.693391* |
| *203* | *Random Forest* | *0.4966887* | *0.675247* |
| *204* | *Random Forest* | *0.5496689* | *0.718322* |
| *205* | *Random Forest* | *0.5165563* | *0.657581* |
| *206* | *Random Forest* | *0.5562914* | *0.700194* |
| *207* | *Random Forest* | *0.4900662* | *0.665567* |
| *208* | *Random Forest* | *0.5364238* | *0.712422* |
| *209* | *Random Forest* | *0.5033113* | *0.659136* |
| *210* | *Random Forest* | *0.5165563* | *0.695075* |
| *211* | *Random Forest* | *0.5298013* | *0.685554* |
| *212* | *Random Forest* | *0.5496689* | *0.715292* |
| *213* | *Random Forest* | *0.5298013* | *0.714267* |
| *214* | *Random Forest* | *0.5231788* | *0.680509* |
| *215* | *Random Forest* | *0.5496689* | *0.698244* |
| *216* | *Random Forest* | *0.5496689* | *0.68776* |
| *217* | *Random Forest* | *0.5033113* | *0.680095* |
| *218* | *Random Forest* | *0.5298013* | *0.682979* |
| *219* | *Random Forest* | *0.5562914* | *0.694037* |
| *220* | *Random Forest* | *0.5562914* | *0.718686* |
| *221* | *Random Forest* | *0.5364238* | *0.647312* |
| *222* | *Random Forest* | *0.5165563* | *0.692277* |
| *223* | *Random Forest* | *0.5430464* | *0.687154* |
| *224* | *Random Forest* | *0.5231788* | *0.702808* |
| *225* | *Random Forest* | *0.5231788* | *0.700878* |
| *226* | *Random Forest* | *0.4900662* | *0.671755* |
| *227* | *Random Forest* | *0.5430464* | *0.687385* |
| *228* | *Random Forest* | *0.5298013* | *0.702332* |
| *229* | *Random Forest* | *0.5298013* | *0.674308* |
| *230* | *Random Forest* | *0.5364238* | *0.70535* |
| *231* | *Random Forest* | *0.5033113* | *0.689878* |
| *232* | *Random Forest* | *0.5033113* | *0.689251* |
| *233* | *Random Forest* | *0.5364238* | *0.659756* |
| *234* | *Random Forest* | *0.5562914* | *0.682057* |
| *235* | *Random Forest* | *0.5099338* | *0.713487* |
| *236* | *Random Forest* | *0.5033113* | *0.691228* |
| *237* | *Random Forest* | *0.5298013* | *0.72388* |
| *238* | *Random Forest* | *0.5231788* | *0.705183* |
| *239* | *Random Forest* | *0.5231788* | *0.679628* |
| *240* | *Random Forest* | *0.589404* | *0.713437* |
| *241* | *Random Forest* | *0.5629139* | *0.707272* |
| *242* | *Random Forest* | *0.5496689* | *0.704109* |
| *243* | *Random Forest* | *0.5827815* | *0.719001* |
| *244* | *Random Forest* | *0.4701987* | *0.64842* |
| *245* | *Random Forest* | *0.4503311* | *0.656344* |
| *246* | *Random Forest* | *0.5364238* | *0.680155* |
| *247* | *Random Forest* | *0.5298013* | *0.694564* |
| *248* | *Random Forest* | *0.5298013* | *0.72711* |
| *249* | *Random Forest* | *0.5231788* | *0.705522* |
| *250* | *Neural Net* | *0.4503311* | *0.658086* |
| *251* | *Neural Net* | *0.4635762* | *0.631774* |
| *252* | *Neural Net* | *0.4635762* | *0.69013* |
| *253* | *Neural Net* | *0.5231788* | *0.702276* |
| *254* | *Neural Net* | *0.4768212* | *0.68158* |
| *255* | *Neural Net* | *0.5099338* | *0.713844* |
| *256* | *Neural Net* | *0.4966887* | *0.691818* |
| *257* | *Neural Net* | *0.4503311* | *0.653552* |
| *258* | *Neural Net* | *0.5364238* | *0.729309* |
| *259* | *Neural Net* | *0.4635762* | *0.676403* |
| *260* | *Neural Net* | *0.5364238* | *0.721531* |
| *261* | *Neural Net* | *0.4966887* | *0.675478* |
| *262* | *Neural Net* | *0.4701987* | *0.658504* |
| *263* | *Neural Net* | *0.4569536* | *0.663001* |
| *264* | *Neural Net* | *0.4966887* | *0.699891* |
| *265* | *Neural Net* | *0.5099338* | *0.71919* |
| *266* | *Neural Net* | *0.4569536* | *0.672271* |
| *267* | *Neural Net* | *0.4966887* | *0.624843* |
| *268* | *Neural Net* | *0.5165563* | *0.715939* |
| *269* | *Neural Net* | *0.4569536* | *0.64881* |
| *270* | *Neural Net* | *0.4900662* | *0.674985* |
| *271* | *Neural Net* | *0.4834437* | *0.673508* |
| *272* | *Neural Net* | *0.5099338* | *0.702368* |
| *273* | *Neural Net* | *0.4900662* | *0.677727* |
| *274* | *Neural Net* | *0.4834437* | *0.659101* |
| *275* | *Neural Net* | *0.5165563* | *0.701558* |
| *276* | *Neural Net* | *0.4768212* | *0.649914* |
| *277* | *Neural Net* | *0.5033113* | *0.705535* |
| *278* | *Neural Net* | *0.5496689* | *0.706725* |
| *279* | *Neural Net* | *0.4635762* | *0.636805* |
| *280* | *Neural Net* | *0.410596* | *0.654073* |
| *281* | *Neural Net* | *0.4900662* | *0.68386* |
| *282* | *Neural Net* | *0.4635762* | *0.649313* |
| *283* | *Neural Net* | *0.5231788* | *0.694799* |
| *284* | *Neural Net* | *0.4768212* | *0.678534* |
| *285* | *Neural Net* | *0.5033113* | *0.695955* |
| *286* | *Neural Net* | *0.4503311* | *0.663173* |
| *287* | *Neural Net* | *0.4900662* | *0.687204* |
| *288* | *Neural Net* | *0.4569536* | *0.654119* |
| *289* | *Neural Net* | *0.4900662* | *0.706339* |
| *290* | *Neural Net* | *0.4304636* | *0.648214* |
| *291* | *Neural Net* | *0.5033113* | *0.646671* |
| *292* | *Neural Net* | *0.5231788* | *0.650725* |
| *293* | *Neural Net* | *0.5099338* | *0.673761* |
| *294* | *Neural Net* | *0.4768212* | *0.677878* |
| *295* | *Neural Net* | *0.5033113* | *0.688209* |
| *296* | *Neural Net* | *0.4569536* | *0.624888* |
| *297* | *Neural Net* | *0.4900662* | *0.689561* |
| *298* | *Neural Net* | *0.5298013* | *0.682829* |
| *299* | *Neural Net* | *0.4900662* | *0.689709* |
| *300* | *AdaBoost* | *0.4437086* | *0.630927* |
| *301* | *AdaBoost* | *0.4701987* | *0.657218* |
| *302* | *AdaBoost* | *0.4039735* | *0.648462* |
| *303* | *AdaBoost* | *0.4635762* | *0.570231* |
| *304* | *AdaBoost* | *0.5033113* | *0.657206* |
| *305* | *AdaBoost* | *0.4304636* | *0.646113* |
| *306* | *AdaBoost* | *0.4966887* | *0.639849* |
| *307* | *AdaBoost* | *0.4701987* | *0.619055* |
| *308* | *AdaBoost* | *0.5099338* | *0.649974* |
| *309* | *AdaBoost* | *0.4768212* | *0.614475* |
| *310* | *AdaBoost* | *0.5364238* | *0.660049* |
| *311* | *AdaBoost* | *0.5496689* | *0.686769* |
| *312* | *AdaBoost* | *0.5430464* | *0.653922* |
| *313* | *AdaBoost* | *0.4701987* | *0.633049* |
| *314* | *AdaBoost* | *0.4569536* | *0.665188* |
| *315* | *AdaBoost* | *0.4238411* | *0.62093* |
| *316* | *AdaBoost* | *0.5033113* | *0.615769* |
| *317* | *AdaBoost* | *0.5298013* | *0.686177* |
| *318* | *AdaBoost* | *0.4569536* | *0.615528* |
| *319* | *AdaBoost* | *0.5099338* | *0.615753* |
| *320* | *AdaBoost* | *0.4635762* | *0.593641* |
| *321* | *AdaBoost* | *0.4701987* | *0.633112* |
| *322* | *AdaBoost* | *0.4437086* | *0.630038* |
| *323* | *AdaBoost* | *0.5099338* | *0.651648* |
| *324* | *AdaBoost* | *0.4701987* | *0.59816* |
| *325* | *AdaBoost* | *0.3708609* | *0.600746* |
| *326* | *AdaBoost* | *0.4437086* | *0.638468* |
| *327* | *AdaBoost* | *0.4370861* | *0.636724* |
| *328* | *AdaBoost* | *0.4834437* | *0.642773* |
| *329* | *AdaBoost* | *0.4039735* | *0.590236* |
| *330* | *AdaBoost* | *0.4304636* | *0.617022* |
| *331* | *AdaBoost* | *0.4238411* | *0.616402* |
| *332* | *AdaBoost* | *0.4701987* | *0.644854* |
| *333* | *AdaBoost* | *0.4900662* | *0.663885* |
| *334* | *AdaBoost* | *0.4569536* | *0.633725* |
| *335* | *AdaBoost* | *0.4701987* | *0.618898* |
| *336* | *AdaBoost* | *0.384106* | *0.611295* |
| *337* | *AdaBoost* | *0.4635762* | *0.611389* |
| *338* | *AdaBoost* | *0.4172185* | *0.638393* |
| *339* | *AdaBoost* | *0.4569536* | *0.626129* |
| *340* | *AdaBoost* | *0.5298013* | *0.626658* |
| *341* | *AdaBoost* | *0.4569536* | *0.626846* |
| *342* | *AdaBoost* | *0.4437086* | *0.617857* |
| *343* | *AdaBoost* | *0.4569536* | *0.609031* |
| *344* | *AdaBoost* | *0.4039735* | *0.581627* |
| *345* | *AdaBoost* | *0.4635762* | *0.660466* |
| *346* | *AdaBoost* | *0.4701987* | *0.622348* |
| *347* | *AdaBoost* | *0.4238411* | *0.613895* |
| *348* | *AdaBoost* | *0.4304636* | *0.606124* |
| *349* | *AdaBoost* | *0.4635762* | *0.600031* |
| *350* | *Naive Bayes* | *0.4172185* | *0.663856* |
| *351* | *Naive Bayes* | *0.4569536* | *0.705683* |
| *352* | *Naive Bayes* | *0.4370861* | *0.68884* |
| *353* | *Naive Bayes* | *0.4900662* | *0.740099* |
| *354* | *Naive Bayes* | *0.4768212* | *0.688868* |
| *355* | *Naive Bayes* | *0.4768212* | *0.70385* |
| *356* | *Naive Bayes* | *0.4569536* | *0.677076* |
| *357* | *Naive Bayes* | *0.4768212* | *0.700397* |
| *358* | *Naive Bayes* | *0.4635762* | *0.686372* |
| *359* | *Naive Bayes* | *0.4039735* | *0.667735* |
| *360* | *Naive Bayes* | *0.4569536* | *0.689653* |
| *361* | *Naive Bayes* | *0.4701987* | *0.694483* |
| *362* | *Naive Bayes* | *0.4966887* | *0.720366* |
| *363* | *Naive Bayes* | *0.5099338* | *0.731843* |
| *364* | *Naive Bayes* | *0.4834437* | *0.686865* |
| *365* | *Naive Bayes* | *0.5298013* | *0.719926* |
| *366* | *Naive Bayes* | *0.4768212* | *0.723397* |
| *367* | *Naive Bayes* | *0.4370861* | *0.691663* |
| *368* | *Naive Bayes* | *0.4966887* | *0.691543* |
| *369* | *Naive Bayes* | *0.4900662* | *0.715673* |
| *370* | *Naive Bayes* | *0.4569536* | *0.697496* |
| *371* | *Naive Bayes* | *0.4900662* | *0.721464* |
| *372* | *Naive Bayes* | *0.4900662* | *0.691832* |
| *373* | *Naive Bayes* | *0.4966887* | *0.718797* |
| *374* | *Naive Bayes* | *0.5231788* | *0.72229* |
| *375* | *Naive Bayes* | *0.4900662* | *0.707463* |
| *376* | *Naive Bayes* | *0.4900662* | *0.676353* |
| *377* | *Naive Bayes* | *0.4768212* | *0.705798* |
| *378* | *Naive Bayes* | *0.4569536* | *0.697586* |
| *379* | *Naive Bayes* | *0.5099338* | *0.719094* |
| *380* | *Naive Bayes* | *0.5033113* | *0.744547* |
| *381* | *Naive Bayes* | *0.4900662* | *0.696892* |
| *382* | *Naive Bayes* | *0.4503311* | *0.718251* |
| *383* | *Naive Bayes* | *0.4039735* | *0.681628* |
| *384* | *Naive Bayes* | *0.4834437* | *0.684787* |
| *385* | *Naive Bayes* | *0.410596* | *0.638053* |
| *386* | *Naive Bayes* | *0.4635762* | *0.671712* |
| *387* | *Naive Bayes* | *0.5562914* | *0.740743* |
| *388* | *Naive Bayes* | *0.5231788* | *0.730098* |
| *389* | *Naive Bayes* | *0.4503311* | *0.685409* |
| *390* | *Naive Bayes* | *0.4370861* | *0.71582* |
| *391* | *Naive Bayes* | *0.5231788* | *0.712* |
| *392* | *Naive Bayes* | *0.4569536* | *0.700485* |
| *393* | *Naive Bayes* | *0.4238411* | *0.664105* |
| *394* | *Naive Bayes* | *0.4768212* | *0.682015* |
| *395* | *Naive Bayes* | *0.5298013* | *0.719099* |
| *396* | *Naive Bayes* | *0.4834437* | *0.691229* |
| *397* | *Naive Bayes* | *0.4834437* | *0.719775* |
| *398* | *Naive Bayes* | *0.4966887* | *0.704121* |
| *399* | *Naive Bayes* | *0.5430464* | *0.713874* |

| Table 3. Fifty-times iteration of models for Fibrosis Stage. | | | |
| --- | --- | --- | --- |
| Column1 | **ClfName** | **Accuracy** | **AUC** |
| 0 | Nearest Neighbors | 0.5827815 | 0.495307 |
| 1 | Nearest Neighbors | 0.5298013 | 0.538308 |
| 2 | Nearest Neighbors | 0.5430464 | 0.494268 |
| 3 | Nearest Neighbors | 0.5496689 | 0.512899 |
| 4 | Nearest Neighbors | 0.5430464 | 0.518774 |
| 5 | Nearest Neighbors | 0.5827815 | 0.550376 |
| 6 | Nearest Neighbors | 0.5430464 | 0.497932 |
| 7 | Nearest Neighbors | 0.5496689 | 0.54399 |
| 8 | Nearest Neighbors | 0.5695364 | 0.506519 |
| 9 | Nearest Neighbors | 0.5496689 | 0.532364 |
| 10 | Nearest Neighbors | 0.589404 | 0.572727 |
| 11 | Nearest Neighbors | 0.5430464 | 0.489554 |
| 12 | Nearest Neighbors | 0.5960265 | 0.535705 |
| 13 | Nearest Neighbors | 0.4900662 | 0.514298 |
| 14 | Nearest Neighbors | 0.5099338 | 0.516429 |
| 15 | Nearest Neighbors | 0.5298013 | 0.533781 |
| 16 | Nearest Neighbors | 0.4834437 | 0.505512 |
| 17 | Nearest Neighbors | 0.5033113 | 0.515059 |
| 18 | Nearest Neighbors | 0.5099338 | 0.480615 |
| 19 | Nearest Neighbors | 0.5430464 | 0.542223 |
| 20 | Nearest Neighbors | 0.5231788 | 0.50716 |
| 21 | Nearest Neighbors | 0.5695364 | 0.563823 |
| 22 | Nearest Neighbors | 0.5364238 | 0.54134 |
| 23 | Nearest Neighbors | 0.5099338 | 0.511108 |
| 24 | Nearest Neighbors | 0.5099338 | 0.500663 |
| 25 | Nearest Neighbors | 0.5033113 | 0.493769 |
| 26 | Nearest Neighbors | 0.5364238 | 0.540875 |
| 27 | Nearest Neighbors | 0.5827815 | 0.532512 |
| 28 | Nearest Neighbors | 0.5496689 | 0.53964 |
| 29 | Nearest Neighbors | 0.5960265 | 0.538674 |
| 30 | Nearest Neighbors | 0.5165563 | 0.535154 |
| 31 | Nearest Neighbors | 0.5430464 | 0.537931 |
| 32 | Nearest Neighbors | 0.5496689 | 0.529191 |
| 33 | Nearest Neighbors | 0.5430464 | 0.539409 |
| 34 | Nearest Neighbors | 0.5364238 | 0.559052 |
| 35 | Nearest Neighbors | 0.6092715 | 0.552347 |
| 36 | Nearest Neighbors | 0.5496689 | 0.534167 |
| 37 | Nearest Neighbors | 0.5033113 | 0.511068 |
| 38 | Nearest Neighbors | 0.5562914 | 0.527925 |
| 39 | Nearest Neighbors | 0.4966887 | 0.487219 |
| 40 | Nearest Neighbors | 0.5827815 | 0.553749 |
| 41 | Nearest Neighbors | 0.5695364 | 0.544285 |
| 42 | Nearest Neighbors | 0.5562914 | 0.527279 |
| 43 | Nearest Neighbors | 0.5695364 | 0.514725 |
| 44 | Nearest Neighbors | 0.5231788 | 0.527206 |
| 45 | Nearest Neighbors | 0.5761589 | 0.544377 |
| 46 | Nearest Neighbors | 0.5629139 | 0.543781 |
| 47 | Nearest Neighbors | 0.5364238 | 0.509928 |
| 48 | Nearest Neighbors | 0.5231788 | 0.53507 |
| 49 | Nearest Neighbors | 0.5562914 | 0.530313 |
| 50 | Linear SVM | 0.5562914 | 0.617593 |
| 51 | Linear SVM | 0.5562914 | 0.648619 |
| 52 | Linear SVM | 0.5695364 | 0.572339 |
| 53 | Linear SVM | 0.5695364 | 0.637712 |
| 54 | Linear SVM | 0.5695364 | 0.62776 |
| 55 | Linear SVM | 0.5562914 | 0.698667 |
| 56 | Linear SVM | 0.5629139 | 0.657063 |
| 57 | Linear SVM | 0.5629139 | 0.593417 |
| 58 | Linear SVM | 0.5562914 | 0.631538 |
| 59 | Linear SVM | 0.5629139 | 0.627618 |
| 60 | Linear SVM | 0.5761589 | 0.661974 |
| 61 | Linear SVM | 0.5629139 | 0.607374 |
| 62 | Linear SVM | 0.5629139 | 0.625736 |
| 63 | Linear SVM | 0.5695364 | 0.594193 |
| 64 | Linear SVM | 0.5629139 | 0.636794 |
| 65 | Linear SVM | 0.5629139 | 0.643752 |
| 66 | Linear SVM | 0.5761589 | 0.570647 |
| 67 | Linear SVM | 0.5695364 | 0.595064 |
| 68 | Linear SVM | 0.5695364 | 0.578377 |
| 69 | Linear SVM | 0.5629139 | 0.620374 |
| 70 | Linear SVM | 0.5629139 | 0.581265 |
| 71 | Linear SVM | 0.5629139 | 0.596985 |
| 72 | Linear SVM | 0.5695364 | 0.54916 |
| 73 | Linear SVM | 0.5695364 | 0.601423 |
| 74 | Linear SVM | 0.5695364 | 0.605044 |
| 75 | Linear SVM | 0.589404 | 0.579091 |
| 76 | Linear SVM | 0.5695364 | 0.570373 |
| 77 | Linear SVM | 0.5695364 | 0.595165 |
| 78 | Linear SVM | 0.5562914 | 0.546607 |
| 79 | Linear SVM | 0.5695364 | 0.646925 |
| 80 | Linear SVM | 0.5629139 | 0.507798 |
| 81 | Linear SVM | 0.5629139 | 0.602294 |
| 82 | Linear SVM | 0.5695364 | 0.607407 |
| 83 | Linear SVM | 0.5562914 | 0.647119 |
| 84 | Linear SVM | 0.5827815 | 0.552773 |
| 85 | Linear SVM | 0.5629139 | 0.628768 |
| 86 | Linear SVM | 0.5629139 | 0.566505 |
| 87 | Linear SVM | 0.5629139 | 0.630651 |
| 88 | Linear SVM | 0.5629139 | 0.636436 |
| 89 | Linear SVM | 0.5629139 | 0.594342 |
| 90 | Linear SVM | 0.5629139 | 0.606789 |
| 91 | Linear SVM | 0.5562914 | 0.631659 |
| 92 | Linear SVM | 0.5629139 | 0.60009 |
| 93 | Linear SVM | 0.5695364 | 0.580773 |
| 94 | Linear SVM | 0.5629139 | 0.620165 |
| 95 | Linear SVM | 0.5629139 | 0.609013 |
| 96 | Linear SVM | 0.5629139 | 0.679816 |
| 97 | Linear SVM | 0.5695364 | 0.56171 |
| 98 | Linear SVM | 0.5629139 | 0.650969 |
| 99 | Linear SVM | 0.5629139 | 0.624316 |
| 100 | RBF SVM | 0.5629139 | 0.635821 |
| 101 | RBF SVM | 0.5629139 | 0.576107 |
| 102 | RBF SVM | 0.5629139 | 0.622634 |
| 103 | RBF SVM | 0.5629139 | 0.632882 |
| 104 | RBF SVM | 0.5629139 | 0.557522 |
| 105 | RBF SVM | 0.5629139 | 0.601696 |
| 106 | RBF SVM | 0.5629139 | 0.614868 |
| 107 | RBF SVM | 0.5629139 | 0.587001 |
| 108 | RBF SVM | 0.5629139 | 0.581174 |
| 109 | RBF SVM | 0.5629139 | 0.583492 |
| 110 | RBF SVM | 0.5629139 | 0.605838 |
| 111 | RBF SVM | 0.5629139 | 0.629616 |
| 112 | RBF SVM | 0.5629139 | 0.653508 |
| 113 | RBF SVM | 0.5629139 | 0.606414 |
| 114 | RBF SVM | 0.5629139 | 0.626077 |
| 115 | RBF SVM | 0.5629139 | 0.583307 |
| 116 | RBF SVM | 0.5562914 | 0.563082 |
| 117 | RBF SVM | 0.5629139 | 0.600542 |
| 118 | RBF SVM | 0.5629139 | 0.623914 |
| 119 | RBF SVM | 0.5629139 | 0.625143 |
| 120 | RBF SVM | 0.5629139 | 0.601698 |
| 121 | RBF SVM | 0.5629139 | 0.626578 |
| 122 | RBF SVM | 0.5629139 | 0.639584 |
| 123 | RBF SVM | 0.5629139 | 0.539417 |
| 124 | RBF SVM | 0.5629139 | 0.636876 |
| 125 | RBF SVM | 0.5629139 | 0.60453 |
| 126 | RBF SVM | 0.5629139 | 0.541562 |
| 127 | RBF SVM | 0.5629139 | 0.617509 |
| 128 | RBF SVM | 0.5629139 | 0.645828 |
| 129 | RBF SVM | 0.5629139 | 0.642227 |
| 130 | RBF SVM | 0.5629139 | 0.551656 |
| 131 | RBF SVM | 0.5629139 | 0.601077 |
| 132 | RBF SVM | 0.5629139 | 0.590681 |
| 133 | RBF SVM | 0.5629139 | 0.596046 |
| 134 | RBF SVM | 0.5629139 | 0.60555 |
| 135 | RBF SVM | 0.5629139 | 0.604239 |
| 136 | RBF SVM | 0.5629139 | 0.640662 |
| 137 | RBF SVM | 0.5629139 | 0.578121 |
| 138 | RBF SVM | 0.5496689 | 0.554028 |
| 139 | RBF SVM | 0.5629139 | 0.585859 |
| 140 | RBF SVM | 0.5629139 | 0.620262 |
| 141 | RBF SVM | 0.5695364 | 0.577152 |
| 142 | RBF SVM | 0.5629139 | 0.614133 |
| 143 | RBF SVM | 0.5629139 | 0.614788 |
| 144 | RBF SVM | 0.5629139 | 0.660312 |
| 145 | RBF SVM | 0.5695364 | 0.593498 |
| 146 | RBF SVM | 0.5629139 | 0.569799 |
| 147 | RBF SVM | 0.5629139 | 0.636464 |
| 148 | RBF SVM | 0.5629139 | 0.630875 |
| 149 | RBF SVM | 0.5629139 | 0.593744 |
| 150 | Gaussian Process | 0.5629139 | 0.601121 |
| 151 | Gaussian Process | 0.5629139 | 0.570965 |
| 152 | Gaussian Process | 0.5629139 | 0.588492 |
| 153 | Gaussian Process | 0.5629139 | 0.619754 |
| 154 | Gaussian Process | 0.5629139 | 0.667792 |
| 155 | Gaussian Process | 0.5629139 | 0.62428 |
| 156 | Gaussian Process | 0.5629139 | 0.545417 |
| 157 | Gaussian Process | 0.5629139 | 0.567864 |
| 158 | Gaussian Process | 0.5629139 | 0.599652 |
| 159 | Gaussian Process | 0.5629139 | 0.5022 |
| 160 | Gaussian Process | 0.5629139 | 0.618759 |
| 161 | Gaussian Process | 0.5629139 | 0.660303 |
| 162 | Gaussian Process | 0.5629139 | 0.603938 |
| 163 | Gaussian Process | 0.5629139 | 0.665303 |
| 164 | Gaussian Process | 0.5629139 | 0.509161 |
| 165 | Gaussian Process | 0.5629139 | 0.575817 |
| 166 | Gaussian Process | 0.5695364 | 0.643563 |
| 167 | Gaussian Process | 0.5629139 | 0.630693 |
| 168 | Gaussian Process | 0.5629139 | 0.640633 |
| 169 | Gaussian Process | 0.5629139 | 0.548015 |
| 170 | Gaussian Process | 0.5827815 | 0.614403 |
| 171 | Gaussian Process | 0.5629139 | 0.595171 |
| 172 | Gaussian Process | 0.5695364 | 0.618655 |
| 173 | Gaussian Process | 0.5629139 | 0.671695 |
| 174 | Gaussian Process | 0.5629139 | 0.644645 |
| 175 | Gaussian Process | 0.5827815 | 0.568812 |
| 176 | Gaussian Process | 0.5629139 | 0.549995 |
| 177 | Gaussian Process | 0.5827815 | 0.628599 |
| 178 | Gaussian Process | 0.5629139 | 0.576703 |
| 179 | Gaussian Process | 0.5629139 | 0.547685 |
| 180 | Gaussian Process | 0.5629139 | 0.617777 |
| 181 | Gaussian Process | 0.5695364 | 0.592975 |
| 182 | Gaussian Process | 0.5629139 | 0.561924 |
| 183 | Gaussian Process | 0.5629139 | 0.678644 |
| 184 | Gaussian Process | 0.5629139 | 0.595121 |
| 185 | Gaussian Process | 0.5629139 | 0.553377 |
| 186 | Gaussian Process | 0.5960265 | 0.550969 |
| 187 | Gaussian Process | 0.5629139 | 0.605263 |
| 188 | Gaussian Process | 0.5629139 | 0.59819 |
| 189 | Gaussian Process | 0.5695364 | 0.588303 |
| 190 | Gaussian Process | 0.5629139 | 0.641276 |
| 191 | Gaussian Process | 0.589404 | 0.591104 |
| 192 | Gaussian Process | 0.5629139 | 0.614226 |
| 193 | Gaussian Process | 0.5629139 | 0.708088 |
| 194 | Gaussian Process | 0.5629139 | 0.612024 |
| 195 | Gaussian Process | 0.5629139 | 0.692858 |
| 196 | Gaussian Process | 0.5629139 | 0.625142 |
| 197 | Gaussian Process | 0.5827815 | 0.634543 |
| 198 | Gaussian Process | 0.5629139 | 0.588101 |
| 199 | Gaussian Process | 0.5629139 | 0.560616 |
| 200 | Random Forest | 0.602649 | 0.620108 |
| 201 | Random Forest | 0.5827815 | 0.576009 |
| 202 | Random Forest | 0.5695364 | 0.617105 |
| 203 | Random Forest | 0.5562914 | 0.587395 |
| 204 | Random Forest | 0.6092715 | 0.509969 |
| 205 | Random Forest | 0.589404 | 0.550059 |
| 206 | Random Forest | 0.5496689 | 0.590299 |
| 207 | Random Forest | 0.5761589 | 0.626952 |
| 208 | Random Forest | 0.5761589 | 0.612018 |
| 209 | Random Forest | 0.589404 | 0.618319 |
| 210 | Random Forest | 0.5827815 | 0.605773 |
| 211 | Random Forest | 0.5761589 | 0.625529 |
| 212 | Random Forest | 0.5562914 | 0.567638 |
| 213 | Random Forest | 0.5960265 | 0.613054 |
| 214 | Random Forest | 0.5827815 | 0.582939 |
| 215 | Random Forest | 0.589404 | 0.502777 |
| 216 | Random Forest | 0.5562914 | 0.520724 |
| 217 | Random Forest | 0.5827815 | 0.525312 |
| 218 | Random Forest | 0.602649 | 0.596472 |
| 219 | Random Forest | 0.589404 | 0.602592 |
| 220 | Random Forest | 0.5827815 | 0.561995 |
| 221 | Random Forest | 0.5496689 | 0.524321 |
| 222 | Random Forest | 0.5629139 | 0.584378 |
| 223 | Random Forest | 0.5827815 | 0.605783 |
| 224 | Random Forest | 0.5960265 | 0.534118 |
| 225 | Random Forest | 0.5761589 | 0.589338 |
| 226 | Random Forest | 0.6092715 | 0.616114 |
| 227 | Random Forest | 0.5827815 | 0.57269 |
| 228 | Random Forest | 0.6092715 | 0.54942 |
| 229 | Random Forest | 0.5761589 | 0.540803 |
| 230 | Random Forest | 0.5629139 | 0.59672 |
| 231 | Random Forest | 0.5629139 | 0.560557 |
| 232 | Random Forest | 0.589404 | 0.576049 |
| 233 | Random Forest | 0.589404 | 0.550398 |
| 234 | Random Forest | 0.5827815 | 0.501895 |
| 235 | Random Forest | 0.589404 | 0.558548 |
| 236 | Random Forest | 0.5629139 | 0.628084 |
| 237 | Random Forest | 0.5629139 | 0.508644 |
| 238 | Random Forest | 0.5827815 | 0.626562 |
| 239 | Random Forest | 0.5761589 | 0.602848 |
| 240 | Random Forest | 0.5695364 | 0.561923 |
| 241 | Random Forest | 0.5562914 | 0.606438 |
| 242 | Random Forest | 0.5827815 | 0.557627 |
| 243 | Random Forest | 0.5761589 | 0.530746 |
| 244 | Random Forest | 0.5960265 | 0.600547 |
| 245 | Random Forest | 0.5960265 | 0.583751 |
| 246 | Random Forest | 0.5761589 | 0.574517 |
| 247 | Random Forest | 0.589404 | 0.559986 |
| 248 | Random Forest | 0.5562914 | 0.504879 |
| 249 | Random Forest | 0.5761589 | 0.547897 |
| 250 | Neural Net | 0.5695364 | 0.546802 |
| 251 | Neural Net | 0.5364238 | 0.573898 |
| 252 | Neural Net | 0.5165563 | 0.578108 |
| 253 | Neural Net | 0.5364238 | 0.529998 |
| 254 | Neural Net | 0.5827815 | 0.581453 |
| 255 | Neural Net | 0.5033113 | 0.588258 |
| 256 | Neural Net | 0.4834437 | 0.543233 |
| 257 | Neural Net | 0.5496689 | 0.537479 |
| 258 | Neural Net | 0.5099338 | 0.553123 |
| 259 | Neural Net | 0.5430464 | 0.639518 |
| 260 | Neural Net | 0.4966887 | 0.579239 |
| 261 | Neural Net | 0.4966887 | 0.53708 |
| 262 | Neural Net | 0.5496689 | 0.617309 |
| 263 | Neural Net | 0.4966887 | 0.595196 |
| 264 | Neural Net | 0.5364238 | 0.58444 |
| 265 | Neural Net | 0.4900662 | 0.55941 |
| 266 | Neural Net | 0.5430464 | 0.605529 |
| 267 | Neural Net | 0.5231788 | 0.590693 |
| 268 | Neural Net | 0.5033113 | 0.556231 |
| 269 | Neural Net | 0.5033113 | 0.628217 |
| 270 | Neural Net | 0.5430464 | 0.60599 |
| 271 | Neural Net | 0.4701987 | 0.589199 |
| 272 | Neural Net | 0.4768212 | 0.608802 |
| 273 | Neural Net | 0.5430464 | 0.562022 |
| 274 | Neural Net | 0.5827815 | 0.625239 |
| 275 | Neural Net | 0.4966887 | 0.508496 |
| 276 | Neural Net | 0.5231788 | 0.583022 |
| 277 | Neural Net | 0.5430464 | 0.589034 |
| 278 | Neural Net | 0.5364238 | 0.545478 |
| 279 | Neural Net | 0.5364238 | 0.568781 |
| 280 | Neural Net | 0.5496689 | 0.610557 |
| 281 | Neural Net | 0.5231788 | 0.582125 |
| 282 | Neural Net | 0.589404 | 0.572223 |
| 283 | Neural Net | 0.5298013 | 0.520335 |
| 284 | Neural Net | 0.5629139 | 0.6135 |
| 285 | Neural Net | 0.4768212 | 0.596968 |
| 286 | Neural Net | 0.5231788 | 0.566443 |
| 287 | Neural Net | 0.4966887 | 0.577319 |
| 288 | Neural Net | 0.5629139 | 0.55992 |
| 289 | Neural Net | 0.5430464 | 0.550494 |
| 290 | Neural Net | 0.4966887 | 0.589372 |
| 291 | Neural Net | 0.4834437 | 0.547026 |
| 292 | Neural Net | 0.5298013 | 0.542633 |
| 293 | Neural Net | 0.5364238 | 0.589041 |
| 294 | Neural Net | 0.5298013 | 0.527902 |
| 295 | Neural Net | 0.4966887 | 0.555074 |
| 296 | Neural Net | 0.5165563 | 0.543142 |
| 297 | Neural Net | 0.5231788 | 0.591652 |
| 298 | Neural Net | 0.5231788 | 0.573999 |
| 299 | Neural Net | 0.5430464 | 0.594703 |
| 300 | AdaBoost | 0.4834437 | 0.459384 |
| 301 | AdaBoost | 0.3708609 | 0.440803 |
| 302 | AdaBoost | 0.5960265 | 0.440257 |
| 303 | AdaBoost | 0.4834437 | 0.502138 |
| 304 | AdaBoost | 0.4966887 | 0.519623 |
| 305 | AdaBoost | 0.5562914 | 0.518418 |
| 306 | AdaBoost | 0.4834437 | 0.543685 |
| 307 | AdaBoost | 0.4569536 | 0.525409 |
| 308 | AdaBoost | 0.602649 | 0.536028 |
| 309 | AdaBoost | 0.4834437 | 0.535312 |
| 310 | AdaBoost | 0.5496689 | 0.525686 |
| 311 | AdaBoost | 0.6225166 | 0.499194 |
| 312 | AdaBoost | 0.4966887 | 0.607513 |
| 313 | AdaBoost | 0.397351 | 0.515568 |
| 314 | AdaBoost | 0.5562914 | 0.459395 |
| 315 | AdaBoost | 0.4635762 | 0.551361 |
| 316 | AdaBoost | 0.4172185 | 0.555971 |
| 317 | AdaBoost | 0.397351 | 0.372325 |
| 318 | AdaBoost | 0.5165563 | 0.583289 |
| 319 | AdaBoost | 0.4039735 | 0.596669 |
| 320 | AdaBoost | 0.5827815 | 0.504841 |
| 321 | AdaBoost | 0.5033113 | 0.539184 |
| 322 | AdaBoost | 0.4900662 | 0.525898 |
| 323 | AdaBoost | 0.4768212 | 0.432739 |
| 324 | AdaBoost | 0.4039735 | 0.493088 |
| 325 | AdaBoost | 0.3178808 | 0.474651 |
| 326 | AdaBoost | 0.397351 | 0.522696 |
| 327 | AdaBoost | 0.4370861 | 0.48619 |
| 328 | AdaBoost | 0.5165563 | 0.534691 |
| 329 | AdaBoost | 0.4768212 | 0.440133 |
| 330 | AdaBoost | 0.4701987 | 0.614042 |
| 331 | AdaBoost | 0.4635762 | 0.44724 |
| 332 | AdaBoost | 0.4635762 | 0.51986 |
| 333 | AdaBoost | 0.4039735 | 0.467673 |
| 334 | AdaBoost | 0.3509934 | 0.489072 |
| 335 | AdaBoost | 0.4768212 | 0.488899 |
| 336 | AdaBoost | 0.384106 | 0.471799 |
| 337 | AdaBoost | 0.4569536 | 0.456314 |
| 338 | AdaBoost | 0.4900662 | 0.559326 |
| 339 | AdaBoost | 0.384106 | 0.583187 |
| 340 | AdaBoost | 0.3576159 | 0.415001 |
| 341 | AdaBoost | 0.4966887 | 0.450286 |
| 342 | AdaBoost | 0.615894 | 0.517398 |
| 343 | AdaBoost | 0.4370861 | 0.372646 |
| 344 | AdaBoost | 0.4701987 | 0.474491 |
| 345 | AdaBoost | 0.5364238 | 0.520308 |
| 346 | AdaBoost | 0.5562914 | 0.458318 |
| 347 | AdaBoost | 0.602649 | 0.507824 |
| 348 | AdaBoost | 0.4569536 | 0.569714 |
| 349 | AdaBoost | 0.3774834 | 0.509124 |
| 350 | Naive Bayes | 0.4701987 | 0.535916 |
| 351 | Naive Bayes | 0.4503311 | 0.538277 |
| 352 | Naive Bayes | 0.5231788 | 0.549319 |
| 353 | Naive Bayes | 0.2649007 | 0.559648 |
| 354 | Naive Bayes | 0.384106 | 0.572016 |
| 355 | Naive Bayes | 0.4701987 | 0.583318 |
| 356 | Naive Bayes | 0.2582781 | 0.558091 |
| 357 | Naive Bayes | 0.5033113 | 0.589916 |
| 358 | Naive Bayes | 0.2781457 | 0.575214 |
| 359 | Naive Bayes | 0.4635762 | 0.560622 |
| 360 | Naive Bayes | 0.1788079 | 0.664287 |
| 361 | Naive Bayes | 0.4039735 | 0.550202 |
| 362 | Naive Bayes | 0.2913907 | 0.563644 |
| 363 | Naive Bayes | 0.4966887 | 0.594435 |
| 364 | Naive Bayes | 0.4834437 | 0.534024 |
| 365 | Naive Bayes | 0.5165563 | 0.54743 |
| 366 | Naive Bayes | 0.4569536 | 0.553415 |
| 367 | Naive Bayes | 0.5364238 | 0.589398 |
| 368 | Naive Bayes | 0.4039735 | 0.580114 |
| 369 | Naive Bayes | 0.4635762 | 0.591936 |
| 370 | Naive Bayes | 0.4635762 | 0.610738 |
| 371 | Naive Bayes | 0.205298 | 0.564339 |
| 372 | Naive Bayes | 0.2649007 | 0.553832 |
| 373 | Naive Bayes | 0.4635762 | 0.548457 |
| 374 | Naive Bayes | 0.3907285 | 0.535109 |
| 375 | Naive Bayes | 0.4370861 | 0.565692 |
| 376 | Naive Bayes | 0.4635762 | 0.538124 |
| 377 | Naive Bayes | 0.2317881 | 0.578559 |
| 378 | Naive Bayes | 0.4966887 | 0.590917 |
| 379 | Naive Bayes | 0.5165563 | 0.620571 |
| 380 | Naive Bayes | 0.2450331 | 0.543804 |
| 381 | Naive Bayes | 0.4635762 | 0.562363 |
| 382 | Naive Bayes | 0.4701987 | 0.576186 |
| 383 | Naive Bayes | 0.4900662 | 0.596774 |
| 384 | Naive Bayes | 0.4900662 | 0.579041 |
| 385 | Naive Bayes | 0.4900662 | 0.628891 |
| 386 | Naive Bayes | 0.5231788 | 0.557222 |
| 387 | Naive Bayes | 0.5430464 | 0.599834 |
| 388 | Naive Bayes | 0.5165563 | 0.511118 |
| 389 | Naive Bayes | 0.5033113 | 0.574892 |
| 390 | Naive Bayes | 0.4370861 | 0.583773 |
| 391 | Naive Bayes | 0.4834437 | 0.558073 |
| 392 | Naive Bayes | 0.5496689 | 0.529808 |
| 393 | Naive Bayes | 0.5629139 | 0.602079 |
| 394 | Naive Bayes | 0.4569536 | 0.583798 |
| 395 | Naive Bayes | 0.4768212 | 0.548181 |
| 396 | Naive Bayes | 0.410596 | 0.565568 |
| 397 | Naive Bayes | 0.5165563 | 0.540692 |
| 398 | Naive Bayes | 0.4635762 | 0.554651 |
| 399 | Naive Bayes | 0.4304636 | 0.534501 |
